# Cell Cycle Arrest and Apoptosis-Inducing Ability of Benzimidazole Derivatives: Design, Synthesis, Docking, and Biological Evaluation

**DOI:** 10.3390/molecules27206899

**Published:** 2022-10-14

**Authors:** Syed Nazreen, Abdulraheem S. A. Almalki, Serag Eldin I. Elbehairi, Ali A. Shati, Mohammad Y. Alfaifi, Ahmed A. Elhenawy, Nawaf I. Alsenani, Anas Alfarsi, Abdulrahman Alhadhrami, Esam A. Alqurashi, Mohammad Mahboob Alam

**Affiliations:** 1 Department of Chemistry, Faculty of Science, Al-Baha University, Al-Baha 65799, Saudi Arabia; 2 Department of Chemistry, Faculty of Science, Taif University, Taif 21974, Saudi Arabia; 3 Department of Biology, Faculty of Science, King Khalid University, Abha 61421, Saudi Arabia; 4 Cell Culture Laboratory, Egyptian Organization for Biological Products and Vaccines, VACSERA Holding Company, Giza 2311, Egypt; 5 Chemistry Department, Faculty of Science, Al-Azhar University, Nasr City, Cairo 11884, Egypt

**Keywords:** 1,3,4-oxadiazole, benzimidazole, cell cycle arrest, apoptosis, docking

## Abstract

In the current study, new benzimidazole-based 1,3,4-oxadiazole derivatives have been synthesized and characterized by NMR, IR, MS, and elemental analysis. The final compounds were screened for cytotoxicity against MDA-MB-231, SKOV3, and A549 cell lines and EGFR for inhibitory activities. Compounds **10** and **13** were found to be the most active against all the tested cell lines, comparable to doxorubicin, and exhibited significant inhibition on EGFR kinase, with IC_50_ 0.33 and 0.38 μM, respectively, comparable to erlotinib (IC_50_ 0.39 μM). Furthermore, these two compounds effectively suppressed cell cycle progression and induced cell apoptosis in MDA-MB-231, SKOV3, and A549 cell lines. The docking studies revealed that these compounds showed interactions similar to erlotinib at the EGFR site. It can be concluded that the synthesized molecules effectively inhibit EGFR, can arrest the cell cycle, and may trigger apoptosis and therefore, could be used as lead molecules in the development of new anticancer agents targeting EGFR kinase.

## 1. Introduction

Epidermal growth factor receptor (EGFR) is a glycoprotein and belongs to the ErBb family of receptor tyrosine kinases that are involved in signal transduction pathways in normal cells via cell proliferation regulation, progression, and survival [1,2]. The ErBb family consists of four members (EGFR/HER-1/ErbB-1), (HER-2/ErbB-2), (HER-3/ErbB-3), and (HER-4/ErbB-4) [3] and three functional domains namely, the extracellular ligand-binding domain, an intracellular cytoplasmic tyrosine kinase domain, and a transmembrane domain [4]. Ligands, on binding with the extracellular domain of receptors, cause the dimerization of inactive EGFR protein, followed by autophosphorylation, leading to the initiation of chain of intracellular events [5,6]. However upregulation of theses tyrosine kinases causes rapid growth of human tumors in the breast, colon, prostate and lung [7,8]. Thus EGFR tyrosine kinase inhibitors that can inhibit dimerization and autophosphorylation, causing alleviation in EGFR concentration, are considered as a hot topic in oncology.

Different substituted quinazoline derivatives (erlotinib, afatinib, lapatinib) are reported as promising EGFR inhibitors [9,10]. Their poor in vivo activity and drug resistance limits the chemotherapeutic effect of these drugs [11,12]; therefore, development of new EGFR inhibitors incorporating a new bioisosteric heterocyclic scaffold could provide new anticancer drugs. Various heterocyclic compounds, such as pyrimidine [13,14], pyrazoline [15], 1,2,3- triazole [16,17], quinoline [18], benzothiazole [19], and benzimidazole [20,21], have provided an alternative to the quinazoline core in improving the activity and drug resistance of these inhibitors [22].

**Figure 1:**
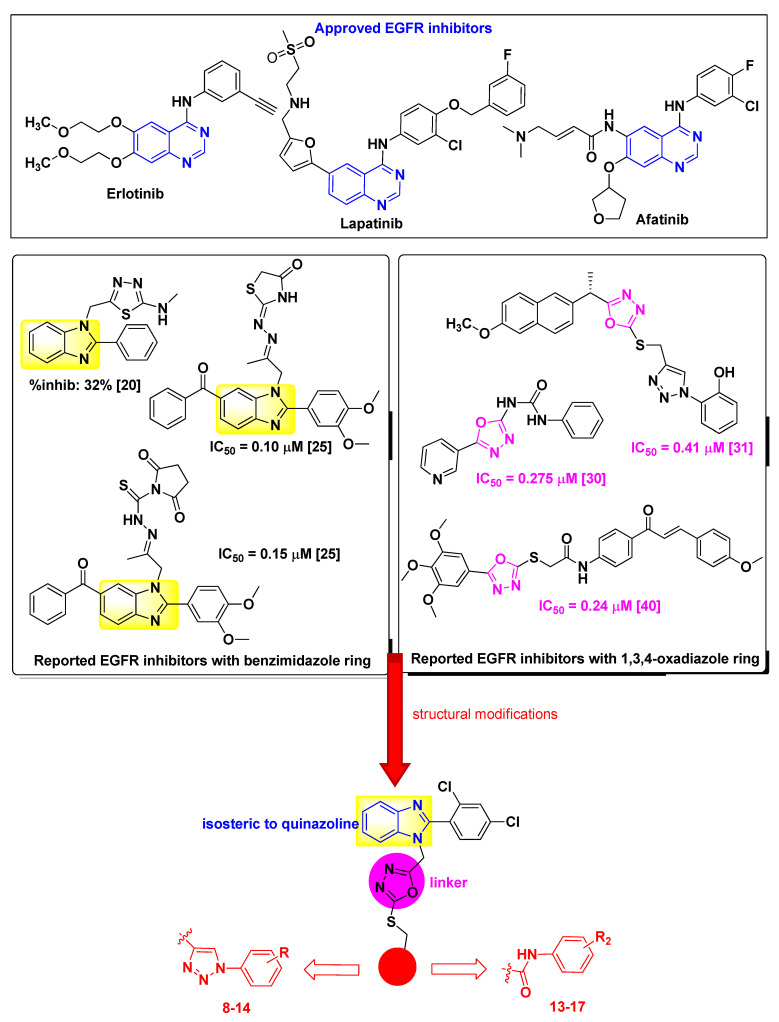
Design of the new benzimidazole based 1,3,4-oxadiazole derivatives as EGFR inhibitors.

In this context benzimidazole, a benzo derivative of imidazole, is an attractive pharmacophore and a master key in medicinal chemistry due to its promising biological activities, including antimicrobial, anti-inflammatory, antitubercular, anticancer, and antidiabetic properties [23,24]. It has been reported that this nitrogen-containing heterocycle has the potential to significantly inhibit EGFR [25] and is present in nazartinib, a third-generation EGFR inhibitor [26]. Therefore, it is an indispensable scaffold for the development of novel chemotherapeutic agents targeting EGFR. On the other hand, 1,3,4-oxadiazole is another important five-membered nitrogen- and oxygen-containing heterocycle due to its significant biological activities [27]. This heterocycle is an effective surrogate in biologically active molecules, and it interacts with biological targets with high affinity, increasing its importance in area of medicinal chemistry [28]. Moreover, this pharmacophore has been reported to inhibit cell proliferation by inhibiting EGFR [29,30,31] (Figure 1). In light of the above evidence, we combined these two moieties together to identify new benzimidazole based 1,3,4-oxadiazole derivatives as cytotoxic agents. The EGFR inhibitory activity and mechanistic investigation on cell cycle distribution and apoptosis studies of the promising molecules were also explored. The docking studies against EGFR protein have been carried out to understand the possible molecular interactions.

## 2. Results and Discussion

### 2.1. Chemistry

**Scheme 1:**
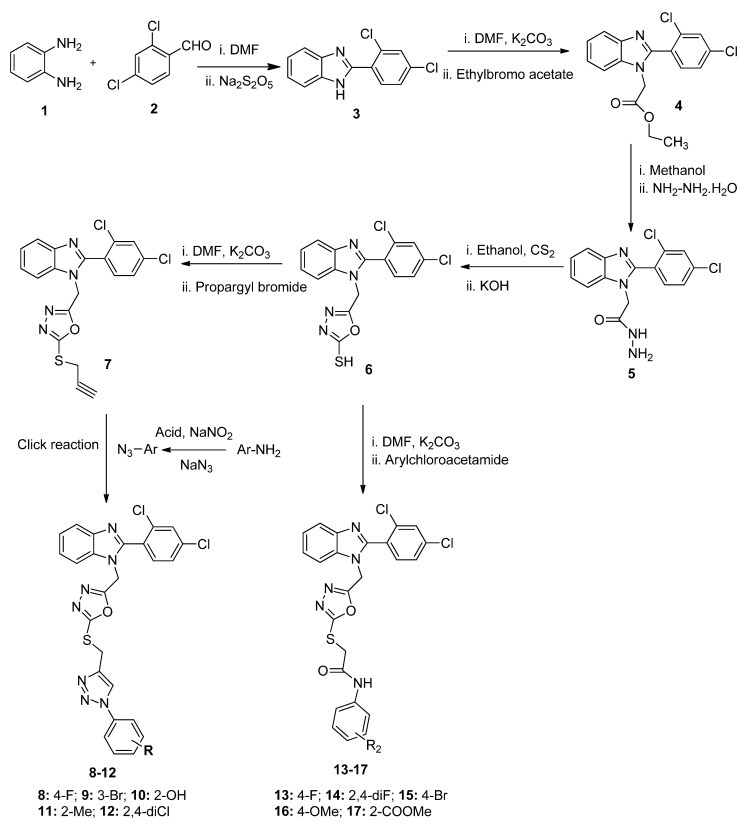
Synthesis of benzimidazole-based 1,3,4-oxadiazole derivatives.

The synthetic method for benzimidazole-based 1,3,4-oxadiazole derivatives is shown in Scheme 1. *O*-phenylenediamine (**1**) was used as a starting material and reacted with 2,4-dichlorobenzaldehyde (**2**) in the presence of sodium metabisulfite (Na_2_S_2_O_5_); using DMF as a solvent in the process resulted in the formation of intermediate (**3**), which upon alkylation with ethyl bromoacetate, followed by treatment with hydrazine hydrate in methanol, yielded compound (**5**). Then, compound (**5**) was reacted with carbon disulfide in alcoholic potassium hydroxide solution, stirred, and refluxed, followed by acidification, which afforded the main intermediate (**6**), which was used for the preparation of the final compounds. The reaction of compound (**6**) with propargyl bromide in DMF and anhydrous potassium carbonate yielded N-propargylated benzimidazole derivative (**7**), which, by using the Click chemistry approach using aromatic azide, copper sulphate, and sodium ascorbate in the presence of tertiary butanol and water, yielded new benzimidazole-based 1,3,4-oxadiazole linked 1,2,3-triazole derivatives (**8–12**). Additionally, the main intermediate (**6**) was reacted with different chloroacetamides in DMF and potassium carbonate to yield new benzimidazole-based 1,3,4-oxadiazole linked thioacetamide derivatives (**13–17**). The formation of all the new compounds was confirmed by different analytical techniques, including NMR, FT-IR, elemental analysis, and mass spectrometry. 

All the newly synthesized benzimidazole-based 1,3,4-oxadiazole linked 1,2,3-triazole derivatives (**8–12**) were confirmed by FTIR spectrum by the disappearance of signals at 3200 cm^−1^ for terminal alkyne C-H stretching. Moreover, in ^1^H NMR spectra, the appearance of two peaks in the aliphatic region at δ 4.56–4.70 ppm and δ 5.41–5.44 ppm were assigned to S-CH_2_- and -N-CH_2_- protons, respectively. The appearance of a downfield signal as a singlet at δ 8.07–8.20 ppm for one proton was assigned to the triazole proton. The ^13^C spectra of these compounds also supported their formation by the presence of two signals in the range δ 26.74–26.92 ppm and δ 38.78–38.96 ppm, corresponding to S-**C**H_2_- and N-**C**H_2_- carbons; finally, the formation of these compounds was confirmed by the appearance of a molecular ion peak in their mass spectra. Similarly, all the newly synthesized benzimidazole-based 1,3,4-oxadiazole linked thio aryl acetamide derivatives (**13–17**) were confirmed by the presence of signals at 3129–3189 cm^−1^ and 1700–1603 cm^−1^ in FT-IR spectra for NH and C=O of thio aryl acetamide group. In ^1^H NMR spectra, the presence of two signals at δ 4.05–4.13 ppm and δ 4.72–5.40 ppm were ascribed to S-CH_2_- and N-CH_2_- protons, while in ^13^C MMR, the respective carbons were observed at δ 33.18–33.53 ppm and 39.17–44.80 ppm, while a downfield signal at δ 168.33–169.49 ppm provided evidence for the presence of C=O group in the structure of these compounds. Finally, the formations of the compounds was confirmed by the presence of molecular ion peaks in their mass spectra.

### 2.2. Biological Activity

#### 2.2.1. Cytotoxicity

The antiproliferative activity of the final newly synthesized compounds (**8–17**) was performed using MTT protocol against three cell lines, viz A549 (lung), SKOV3 (ovarian), and MDA-MB-231 (breast) [32]. Doxorubicin was used as a standard anticancer drug. The results regarding anticancer activity are presented as IC_50_ in Table 1. Towards the A549 cells, compounds **10** and **13** were the most promising candidates, exhibiting better cytotoxicity than doxorubicin (IC_50_ 5.85 μM). Compounds **10** (IC_50_ 3.31 μM) and **13** (IC_50_ 5.30 μM) were 1.76- and 1.1- times more active than doxorubicin, while the remaining compounds exhibited moderate cytotoxicity on this cell line, with IC_50_ in the range 11.64–43.80 μM. Against the MDA-MB-231 cancer cell line, the same compounds, **10** and **13,** were found to be most potent in exerting cytotoxicity, with IC_50_ 1.18 (4.0-fold increase) and 2.90 μM (1.64-fold increase), respectively, whereas doxorubicin showed IC_50_ 4.76 μM. Furthermore, compounds **10** and **13** were also the most sensitive towards the ovarian SKOV3 cell line, displaying IC_50_ 6.98 μM and 4.35 μM, 1.23- and 1.98-fold better activity, respectively, than doxorubicin (IC_50_ 8.65 μM) while compound **16** (IC_50_ 8.13 μM) was equipotent to doxorubicin. Other compounds showed moderate cytotoxicity, with IC_50_ in the range 10.49–36.74 μM and 12.18–33.60 μM on MDA-MB-231 and SKOV3 cell lines, respectively. From these results, it was observed that compound **10,** bearing 2-hydroxy (from 1,3,4-oxadiazole linked 1, 2, 3-triazole series) and compound, **13** bearing 4F (from 1,3,4-oxadiazole linked thioacetamide series), were found to be the most active against all the tested cell lines. 

#### 2.2.2. In Vitro EGFR Activity

Compounds **9, 10, 13, 14, 16,** and **17,** showing significant cytotoxicity on the tested cancerous cell lines, were selected for testing regarding EGFR inhibitory activity to determine the mechanisms of action of these compounds. The results are shown in Table 2 and compared with the standard drug erlotinib. Compounds **10** and **13** significantly inhibited EGFR kinase, with IC_50_ 0.33 μM and 0.38 μM, respectively, while erlotinib caused inhibition with IC_50_ 0.39 μM. Compounds **9, 14, 16,** and **17** also caused moderate EGFR inhibition, with IC_50_ in the range 0.95–1.54 μM. These data suggest that the synthesized compounds exhibited antiproliferative activity via alleviating EGFR kinase.

#### 2.2.3. Cell Cycle Studies

**Figure 2:**
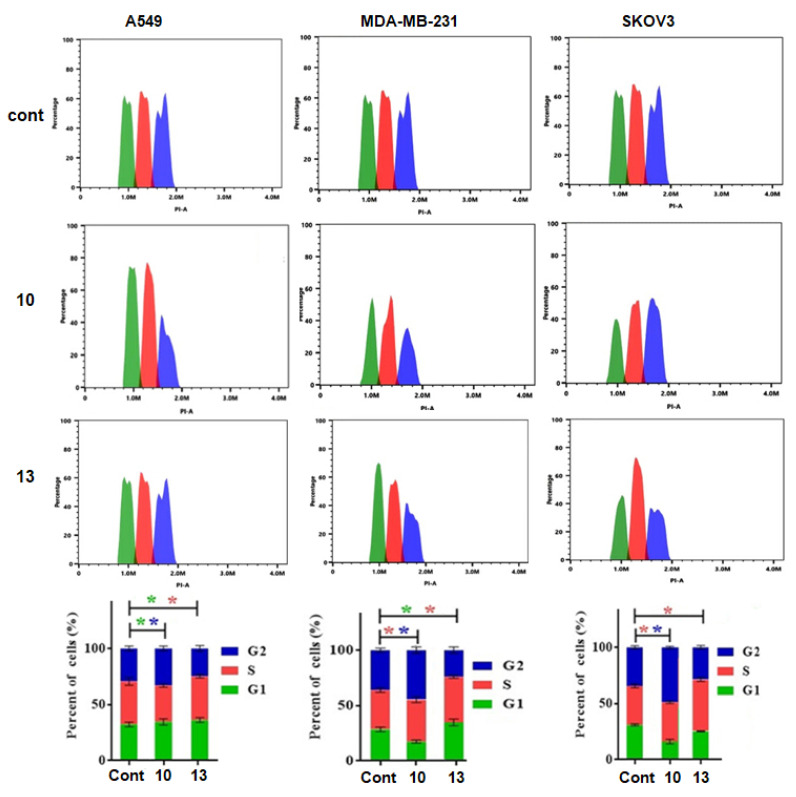
Cell cycle distribution of A549, MDA-MB-231, and SKOV3 cancer cells after treatment with vehicle control and compounds **10** and **13** by propidium iodide staining using flow cytometry. One-way ANOVA was used to test for statistical difference (* *p* < 0.05).

The most promising compounds (**10** and **13**) were explored for cell cycle distribution to determine the intracellular mode of action of these compounds. The cells and vehicle control were treated with each pre calculated IC_50_s for 48 h, were stained with propidium iodide, and observed for cell cycle distribution by flow cytometry [33]. As shown in Figure 2, compound **10-**treated A549 cells increased the G1 and G2 phase from 32.88 ± 2.04% to 34.66 ± 2.80% and from 29.13 ± 2.31% to 33.01 ± 2.28%, respectively, but decreased the cell distribution in the S phase from 38.48 ± 3.71% to 32.32 ± 1.51%, compared to the control cells. Compound **13**-treated cells showed an increase in the G1 phase to 36.34 ± 2.19% and a decrease in G2 phase to 24.64 ± 2.52%; however, it caused no appreciable change in cell distribution in the S phase (38.99 ± 1.67%), compared to control cells (38.48 ± 3.71%). These results showed that compound **10** arrests the cell cycle in the G1 and G2 phases, whereas compound **13** arrests the cell cycle in the G1 phase in the A549 cells. In MDA-MB-231 breast cells, compound **13** significantly increased cell distributions in the G1 phase from 28.51 ± 2.09% to 34.88 ± 3.09%, and in the S phase from 35.51 ± 2.10% to 41.14 ± 1.34%, whereas it decreased the G2 cell population to 23.97 ± 2.93% from 35.96 ± 1.97% (Figure 2). Compound **10** caused a prominent increase in cell distribution in the G2 phase to 44.58 ± 3.03%, and in the S phase to 37.82 ± 2.57%; however, the G1 fraction decreased to 17.58 ± 1.45%. It was noted that compound **13** arrested the cell cycle in the G1 and S phases, while compound **10** arrested the cell cycle in the S and G2 phases. In the SKOV3 cells, the S phase increased significantly from 34.72 ± 1.80% in the absence of the drug (vehicle control) to 45.93 ± 1.78% and 35.11 ± 1.21% in the presence of compound **13** and **10,** respectively; the G1 phase decreased to 25.38 ± 0.44% and 16.15 ± 2.11% from 31.03 ± 1.86% in the presence of compounds **13** and **10**, respectively, whereas the G2 phase was also decreased by compound **13** to 28.65 ± 1.73%, but significantly increased to 48.73± 1.89% from 34.23 ± 1.38% by compound **10**. It is well known that erlotinib is a small molecule EGFR inhibitor that inhibits the intracellular phosphorylation of the tyrosine kinase domain of the EGFR, blocking its activity and resulting in cell cycle arrest. Previous studies have shown that erlotinib induces cell cycle arrests at the G0/G1 phase in non-small cell lung cancer (NSCLC) [34], at the G1/S checkpoint in hepatocellular carcinoma [35], and at the G1/G0 phase in esophageal cancer [36], triggering apoptosis in cancer cells. In the present study, compounds **10** and **13** arrest different cell cycle phases in A549, MDA-MB 231, and SKOV3 cancer cells. Compound **13** induces G1/S arrest in both A549 and MDA-MB 231 cells, but only the S phase in SKOV3 cells. However, compound **10** arrests the G2/S phase in both MDA-MB 231 and SKOV3, while it arrests the G1/G2 phases in lung A549 carcinoma. These results indicate that compounds **10** and **13** arrest the cell cycle at the S, G1, and G2 phases, supporting the promising cytotoxicity in A549, MDA-MB-231, and SKOV3 cells.

#### 2.2.4. Apoptosis Studies

**Figure 3:**
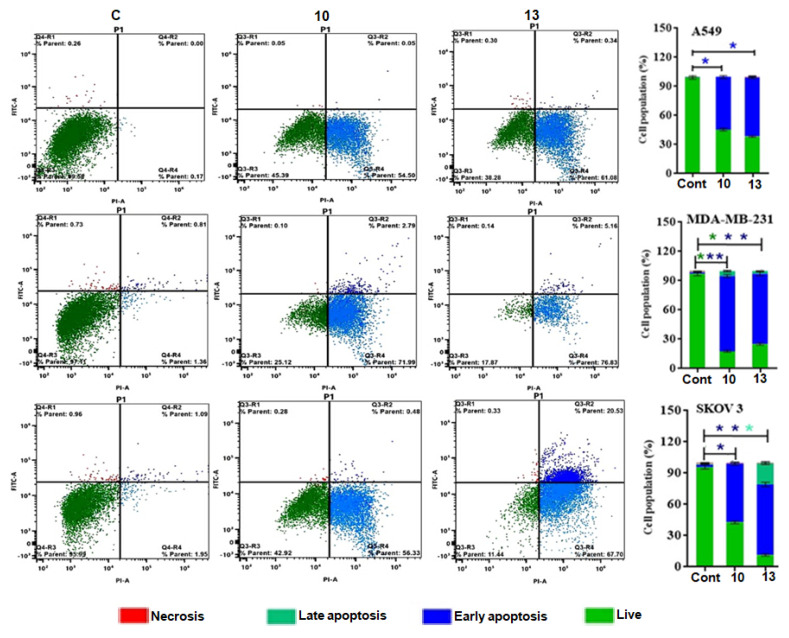
The apoptotic effect in A549, MDA−MB 231, and SKOV3 cancerous cells after treatment with vehicle control and compounds **10** and **13** by annexin V−FITC/PI staining using flow cytometry. One-way ANOVA was used to test for statistical differences (* *p* < 0.05, ** *p* < 0.01).

The apoptosis studies regarding compounds **10** and **13** using annexin V-FITC/PI staining was also examined by flow cytometry [37]. Compounds **10** and **13** caused early apoptosis by 55.1% and 66.4% in A549 cancer cells, with no late apoptosis or necrosis (Figure 3). In MDA-MB-231 cells, compound **13** induced significant early-and late-apoptotic cell populations, increasing them by 80.24% and 5.4%, whereas compound **10** increased early apoptosis by 69.7% and late apoptosis by 3.2%, without any necrosis. Towards SKOV3 ovarian cancer cells, compound **13** caused significant apoptosis increase by 76.2%, for early apoptosis and 21.7%, for late apoptosis. However, compound **10** had a low early apoptotic effect, with an increase of 58.5% in SKOV3 cells. These results suggest that the promising cytotoxicity exhibited by these compounds is due to the induction of apoptosis in the tested cell lines.

### 2.3. Molecular Docking Studies

**Figure 4:**
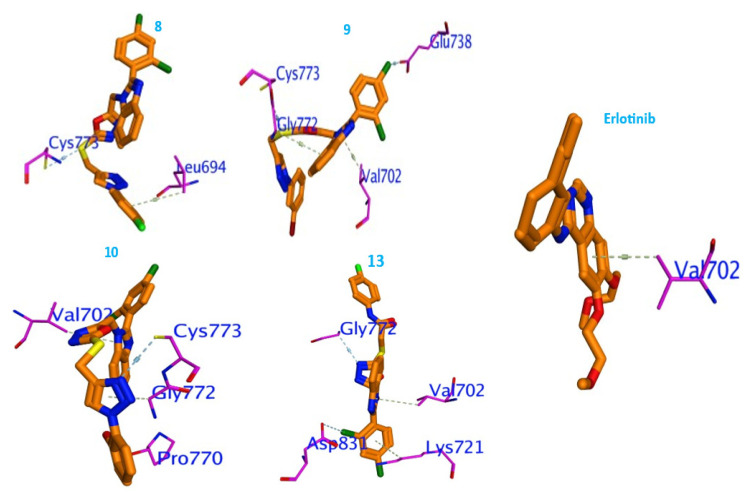
Binding interaction of active compounds **8**, **9**, **10**, **13** and erlotinib against EGFR protein (PDB1M17).

In the present study, the Maestro module [38] was used to utilize the molecular docking into the active site of EGFR (PDB 1M17) to determine the suitable mechanism of anticancer activity for the compounds. For docking, the mGenTHERADER [39] was created and validated by re-docking the original inhibitor, erlotinib, to the EGFR crystal structure. Erlotinib was refitted successfully into the EGFR binding site with 1.94Å RMSD and found to be fitted into the key amino acids (Lys721, Phe699, Val702, Cys773, Leu820, Asn818, Asp783, Asn784, Gln958, Gln962, Met96, Met964, and Leu977). In order to further validate the inhibition mechanism, the bioactivity factors, such as the inhibition constant (Ki), ligand efficiency (LE), and fit quality were also examined [40] (Table 3). The different binding modes for the tested compounds against EGFR (1M17) are represented in Figure 4. The molecular docking showed that all compounds interacted with the EGFR enzyme in the analogous mode to that of erlotinib. The results revealed that the free binding energy of four compounds **8**, **9**, **10**, and **13** (−8.01, −8.16, −8.27, and −8.31 kcal/mol) was higher than that of the cocrystal inhibitor, erlotinib (−7.90 kcal/mol). The reference inhibitor occupied the binding pocket through interaction with Val70. Compounds **8**, **9**, **10,** and **13** caused the inhibition of EGFR kinases by forming an interaction with Cys773 and Leu694; Val702, Gly772, Glu738, and Cys773; Gly772 and Cys773; and Gly772, respectively. The hydrogen bonding interactions in compound **10** and **13** with Val702 and Pro770; and Lys 721, Val702, and Asp831 explained the highest binding affinity and higher potency for these compounds, which suggested that these compounds have a better interaction and antitumor potency than the standard drug. The other tested compounds showed lower interaction energy than the reference erlotinib. All compounds showed a normal range of bioactivity parameters Ki, LE, and FQ [41].

### 2.4. In Silico Toxicity Studies

The in silico toxicity prediction of compounds **10** and **13** were carried out by pkCSM software [42], and the results are shown in Table S1. It was observed that compounds **10** and **13** were less toxic compared to the standard drug erlotinib; the maximum tolerated dose for humans for compounds **10** and **13** was 0.3 and 0.125 mg/kg/day, respectively, which are much higher than the maximum dose for erlotinib (0.002 mg/kg/day). The predicted LD_50_ for rats for compounds **10** and **13** was 2.43 mole/kg and 2.47 mol/kg respectively, slightly higher than for erlotinib (2.368 mol/kg). Moreover, compounds **10** and 13 did not showed hepatotoxicity or skin sensitization, but erlotinib was found to be hepatotoxic. 

## 3. Experimental

### 3.1. Chemistry

#### 3.1.1. General

The chemicals and solvents used were procured from Sigma Aldrich, (St. Louis, MO, USA). The NMR analysis of the synthesized compounds was performed on a Bruker spectrometer, either in DMSOd_6_ or CDCl_3_. FT-IR spectra were obtained using a Thermo Scientific iS 50, and the melting point was obtained using a Stuart SMP40 machine. The mass spectra were obtained using a Thermo Scientific LCQ Fleet- LCF10605 mass spectrometer, and elemental analysis was performed on a LEECO Elementar Analyzer. The intermediates 3-7 were prepared by the reported methods [43]. 

#### 3.1.2. Synthesis of Final Compounds **8–12**

Compounds **8–12** were prepared according to our previously reported work [43]. Compound **7** (2 millimole) was placed in a round-bottom flask to which tertiary butanol:water (20 mL; 1:1) was added to make a clear solution. To this reaction mixture, copper sulphate and sodium ascorbate were added, followed by the addition of freshly prepared aromatic azides. The reaction mixture was stirred until completion of the reaction at 30–60 °C. When the reaction was completed, 100 mL of water was added to the reaction mixture, and the products were extracted with MDC, washed with water, and recrystallized by isopropyl alcohol and ethyl acetate.

2-{[2-(2,4-Dichlorophenyl)-1*H*-benzo[*d*]imidazol-1-yl]methyl}-5-({[1-(4-Fluorophenyl)-1*H*-1,2,3-triazol-4-yl]methyl}thio)-1,3,4-oxadiazole (**8**): Yield: 72%; M.p. 110–112 °C; FT IR: 3026, 2925, 1514, 1471, 1452, 1392, 1232, 1156, 1095, 1044, 834, 747 cm^−1^;^1^H NMR (CDCl_3_, 850 MHz): δ 4.70 (s, 2H, S-CH_2_-), 5.41 (s, 2H, N-CH_2_-), 7.21–7.23 (m, 3H, Ar-H), 7.28 (s, 1H, Ar-H), 7.40–7.71 (m, 5H, Ar-H), 7.84–7.89 (m, 2H, Ar-H), 8.07 (s, 1H, triazole H); ^13^C NMR (CDCl_3_, 213 MHz): δ 26.92, 38.78, 110.11, 116.78, 116.89, 122.66, 122.70, 126.99, 127.99, 133.28, 137.64, 139.69, 139.99, 143.78, 143.89, 161.94, 163.11. ESI MS: 552.67 [M+H]^+^, 554.67 [M+H+2]^+^; C_25_H_16_Cl_2_FN_7_OS (Calcd): C, 54.36; H, 2.92; N, 17.75; S, 5.80. Obsd: C, 54.29; H, 2.91; N, 17.71; S, 5.82.

2-({[1-(3-Bromophenyl)-1*H*-1,2,3-triazol-4-yl]methyl}thio)-5-{[2-(2,4-Dichlorophenyl)-1*H*-benzo[*d*]imidazol-1-yl]methyl}-1,3,4-oxadiazole (**9**): Yield: 84%; M.p. 180–182 °C; FT IR: 2971, 1586, 1452, 1394, 1234, 1166, 1092, 1046, 875, 820, 781, 748, 679 cm^−1^;^1^H NMR (CDCl_3_, 850 MHz): δ 4.56 (s, 2H, S-CH_2_-), 5.44 (s, 2H, N-CH_2_-), 7.28–7.41 (m, 3H, Ar-H), 7.44–7.74 (m, 5H, Ar-H), 7.89–7.94 (m, 3H, Ar-H), 8.14 (s, 1H, triazole H); ^13^C NMR (CDCl_3_, 213 MHz): δ 26.92, 38.78, 110.15, 116.15, 116.43, 119.15, 123.43, 123.73, 128.17, 131.19, 131.94, 132.02, 137.91, 146.17, 149.75, 150.73, 162.11, 165.43. ESI MS: 612.50 [M+H]^+^, 614.50 [M+H+2]^+^; C_25_H_16_BrCl_2_N_7_OS (Calcd): C, 48.96; H, 2.63; N, 15.99; S, 5.23.Obsd: C, 48.84; H, 2.65; N, 15.95; S, 5.21.

2-(4-{[(5-{[2-(2,4-Dichlorophenyl)-1*H*-benzo[*d*]imidazol-1-yl]methyl}-1,3,4-oxadiazol-2-yl)thio]methyl}-1*H*-1,2,3-triazol-1-yl)phenol (**10**): Yield: 78%; M.p. 118-120 °C; FT IR: 2987, 1597, 1472, 1455, 1394, 1232, 1159, 1045, 746 cm^−1^; ^1^H NMR (CDCl_3_, 850 MHz): δ 4.57 (s, 2H, S-CH_2_), 5.41 (s, 2H, N-CH_2_-), 7.01 (d, *J* = 6.8 Hz, 1H, Ar-H), 7.19 (t, *J* = 8.5 Hz, 1H, Ar-H), 7.31 (t, *J* = 7.65 Hz, 1H, Ar-H), 7.36–7.44 (m, 4H, Ar-H), 7.53–7.58 (m, 3H, Ar-H), 7.86 (s, 1H, Ar-H), 8.20 (s, 1H, Ar-H), 9.70 (s, 1H, Ar-OH); ^13^C NMR (CDCl_3_, 213 MHz): δ: 26.92, 38.96, 110.05, 116.02, 118.38, 119.40, 119.99, 120.33, 120.51, 121.12, 123.68, 124.41, 125.35, 127.93, 129.98, 130.04, 133.37, 134.18, 134.99, 137.84, 149.17, 162.11, 165.57; ESI MS: 548.17 [M-H]^+^, 550.17 [M+H+2]^+^; C_25_H_17_Cl_2_N_7_OS (Calcd): C, 54.55; H, 3.11; N, 17.81; S, 5.83.Obsd: C, 54.61; H, 3.14; N, 17.76; S, 5.80.

2-{[2-(2,4-Dichlorophenyl)-1*H*-benzo[*d*]imidazol-1-yl]methyl}-5-({[1-(*o*-tolyl)-1*H*-1,2,3-triazol-4-yl]methyl}thio)-1,3,4-oxadiazole (**11**): Yield 82%; M.p. 152–153 °C; FT IR: 3102, 2979, 1483, 1452, 1347, 1241 1152, 1065, 987, 810, 745 cm^−1^; ^1^H NMR (CDCl_3_, 850 MHz): δ 2.17 (s, 3H, Ar-CH_3_), 4.56 (s, 2H, S-CH_2_-), 5.42 (s, 2H, N-CH_2_-), 7.17–7.71 (m, 10H, Ar-H), 7.84 (brds, 1H, Ar-H), 8.11 (s, 1H, triazole H); ^13^C NMR (CDCl_3_, 213 MHz): δ 17.53, 26.76, 38.83, 111.25, 120.45, 122.67, 124.31, 125.23, 127.14, 127.69, 128.34, 128.37, 128.74, 128.94, 129.19, 130.42, 131.45, 133.74, 136.31, 138.03, 142.11, 162.94, 165.78; ESI MS: 548.75 [M+H]^+^, 550.75 [M+H+2]^+^; C_26_H_19_Cl_2_N_7_OS (Calcd): C, 56.94; H, 3.49; N, 17.88; S, 5.85. Obsd: C, 57.06; H, 3.51; N, 17.94; S, 5.87.

2-({[1-(2,4-Dichlorophenyl)-1*H*-1,2,3-triazol-4-yl]methyl}thio)-5-{[2-(2,4-Dichlorophenyl)-1*H*-benzo[*d*]imidazol-1-yl]methyl}-1,3,4-oxadiazole (**12**): Yield 77%; M.p. 100-102 °C; FT IR: 2970, 1495, 1472, 1453, 1393, 1240, 1161, 1104, 1066, 1041, 986, 866, 813, 746 cm^−1^; ^1^H NMR (CDCl_3_, 850 MHz): δ 4.57 (s, 2H, S-CH_2_-), 5.41 (s, 2H, N-CH_2_-), 7.29–7.63 (m, 9H, Ar-H), 7.84(brds, 1H, Ar-H), 8.12(s, 1H, triazole H); ^13^C NMR (CDCl_3_, 213 MHz): δ 26.74, 38.94, 110.04, 120.44, 123.54, 124.34, 125.74, 127.15, 128.08, 128.35, 128.37, 128.44, 128.47, 129.28, 130.43, 130.55, 133.36, 136.45, 137.71, 142.14, 162.11, 165.67; ESI MS: 602.42 [M+H]^+^, 604.42 [M+H+2]^+^; C_25_H_15_Cl_4_N_7_OS (Calcd): C, 49.77; H, 2.51; N, 16.25; S, 5.31.Obsd: C, 49.68; H, 2.56; N, 16.21; S, 5.28.

#### 3.1.3. Synthesis of Final Compounds **13–17**

Compounds **13–17** were also prepared according to our previously reported method [43]. Compound 6 (1mmol) was placed in a 100 mL round-bottom flask, and 25 mL DMF was added to make a clear solution. To the reaction mixture, anhydrous potassium carbonate (1.3 mmol) was added, followed by the addition of different chloroacetamide derivatives (1.1 mmol). The reaction mixture was stirred at 50–70 °C until completion of the reaction. After the completion of the reaction, the reaction mixture was filtered, cooled, and water (100–150 mL) was added to the filtrate; the products were extracted with MDC. The organic layer was concentrated and recrystallized using either isopropyl alcohol or ethanol.

2-[(5-{[2-(2,4-Dichlorophenyl)-1*H*-benzo[*d*]imidazol-1-yl]methyl}-1,3,4-oxadiazol-2-yl)thio]-N-(4-Fluorophenyl)acetamide (**13**): Yield:71%; M.p. 178-180 °C; FT IR: 3186, 2987, 1683, 1597, 1507, 1455, 1374, 1225, 1155, 1096, 1046, 830, 744 cm^−1^; ^1^H NMR (CDCl_3_, 850 MHz): δ 4.06 (s, 2H, S-CH_2_), 4.72 (s, 2H, N-CH_2_-), 6.98–7.00 (m, 3H, Ar-H), 7.19–7.26 (m, 2H, Ar-H), 7.34–7.35 (m, 2H, Ar-H), 7.40–7.45 (m, 2H, Ar-H), 7.64 (s, 1H, Ar-H), 7.85 (d, *J* = 7.65 Hz, 1H, Ar-H), 8.94 (s, N-H, 1H); ^13^C NMR(CDCl_3_, 213 MHz): δ 33.22, 44.71, 110.06, 115.66, 116.55, 119.95, 120.71, 121.62, 121.66, 123.62, 124.35, 127.91, 127.97, 130.04, 133.34, 133.84, 134.25, 135.03, 137.78, 142.78, 149.60, 162.45, 163.18, 164.73, 169.23; ESI MS: 528.67 [M+H]^+^, 530.67 [M+H+2]^+^; C_24_H_16_Cl_2_FN_5_O_2_S (Calcd): C, 54.55; H, 3.05; N, 13.25; S, 6.07.Obsd: C, 54.51; H, 2.94; N, 13.33; S, 6.02.

2-[(5-{[2-(2,4-Dichlorophenyl)-1*H*-benzo[*d*]imidazol-1-yl]methyl}-1,3,4-oxadiazol-2-yl)thio]-N-(2,4-Difluorophenyl)acetamide (**14**): Yield:79%; M.p. 212–214 °C; FT IR: 3155, 2978, 1740, 1674, 1603, 1514, 1456, 1382, 1331, 1260, 1202, 1096, 1007, 744 cm^−1^; ^1^H NMR (CDCl_3_, 850 MHz): δ 4.05 (s, 2H, S-CH_2_-), 4.76 (s, 2H, N-CH_2_-), 6.77–7.36 (m, 5H, Ar-H), 7.38–7.82 (m, 4H, Ar-H), 7.88 (brd, s, 1H, Ar-H), 8.59 (s, 1H, N-H); ^13^C NMR (CDCl_3_, 213 MHz): δ: 33.19, 44.65, 110.46, 116.65, 120.26, 120.76, 121.37, 123.62, 124.29, 127.57, 127.92, 129.58, 130.71, 133.77, 134.97, 135.40, 136.99, 142,75, 147.37, 148.27, 150.50, 161.26, 164.63, 168.63. ESI MS: 546.67 [M+H]^+^, 548.67 [M+H+2]^+^; C_24_H_15_Cl_2_F_2_N_5_O_2_S (Calcd): C, 52.76; H, 2.77; N, 12.82; S, 5.87. Obsd: C, 52.66; H, 2.85; N, 12.88; S, 5.89.

N-(4-Bromophenyl)-2-[(5-{[2-(2,4-Dichlorophenyl)-1*H*-benzo[d]imidazol-1-yl]methyl}-1,3, 4-oxadiazol-2-yl)thio]acetamide (**15**): Yield: 82%; M.p. 220–222 °C; FT IR: 3186, 2987, 1674, 1603, 1540, 1508, 1488, 1454, 1380, 1257, 1097, 1067, 746 cm^−1^; ^1^H NMR (CDCl_3_, 850 MHz): δ 4.06 (s, 2H, S-CH_2_), 4.73 (s, 2H, N-CH_2_-), 6.79 (d, *J* = 8.5 Hz, 2H, Ar-H), 7.13 (d, *J* = 8.5 Hz, 2H, Ar-H), 7.35–7.84 (m, 6H, Ar-H), 7.89 (s, 1H, Ar-H), 9.14 (s, 1H, -N-H); ^13^C NMR (CDCl_3_, 213 MHz): δ 33.24, 43.09, 109.82, 120.75, 121.37, 122.60, 123.52, 123.61, 124.34, 127.92, 129.09, 129.57, 130.04, 132.02, 132.08, 132.50, 132.74, 133.33, 133.70, 133.81, 151.08, 161.04, 168.33.ESI MS: 588.08 [M+H]^+^, 590.08 [M+H+2]^+^; C_24_H_16_BrCl_2_N_5_O_2_S (Calcd): C, 48.92; H, 2.74; N, 11.88; S, 5.44.Obsd: C, 49.02; H, 2.70; N, 11.75; S, 5.49.

2-[(5-{[2-(2,4-Dichlorophenyl)-1*H*-benzo[*d*]imidazol-1-yl]methyl}-1,3,4-oxadiazol-2-yl)thio]-N-(4-Methoxyphenyl)acetamide (**16**): Yield: 75%; M.p. 195–197 °C; FT IR: 3129, 2987, 1671, 1596, 1508, 1451, 1374, 1248, 1167, 1097, 1041, 826, 747 cm^−1^; ^1^H NMR (CDCl_3_, 850 MHz): δ 3.85 (s, 3H, Ar-O-CH_3_), 4.05 (s, 2H, S-CH_2_), 4.74 (s, 2H, N-CH_2_-), 6.95 (d, *J* = 8.5 Hz, 2H, Ar-H), 7.18 (d, *J* = 8.5 Hz, 2H, Ar-H), 7.28 (d, *J* = 8.5 Hz, 1H, Ar-H), 7.34–7.36 (m, 3H, Ar-H), 7.55–7.58 (m, 2H, Ar-H), 7.87 (s, 1H, Ar-H), 8.18 (s, 1H, N-H); ^13^C NMR (CDCl_3_, 213 MHz): δ 33.18, 44.80, 55.55, 110.11, 114.72, 114.82, 126.24, 127.64, 127.92, 128.43, 128.58, 128.72, 129.61, 133.91, 134.64, 149.13, 160.01, 169.49. ESI MS: 540.83 [M+H]^+^, 542.83 [M+H+2]^+^; C_25_H_19_Cl_2_N_5_O_3_S (Calcd): C, 55.56; H, 3.54; N, 12.96; S, 5.93. Obsd: C, 55.50; H, 3.61; N, 13.05; S, 5.89.

Methyl 2-{2-[(5-{[2-(2,4-Dichlorophenyl)-1*H*-benzo[*d*]imidazol-1-yl]methyl}-1,3,4-oxadia zol -2-yl)thio]acetamido}benzoate (**17**): Yield:71%; M.p. 176–178 °C; FT IR: 3189, 2987, 1700, 1676, 1595, 1521, 1481, 1452, 1394, 1255, 1184, 1089, 1046, 745 cm^−1^;^1^H NMR (CDCl_3_, 850 MHz): δ 3.83 (s, 3H, O-CH_3_), 4.13 (s, 2H, S-CH_2_-), 5.40 (s, 2H, N-CH_2_), 7.13 (t, *J* = 8.5 Hz, 1H, Ar-H), 7.32–7.59 (m, 7H, Ar-H), 7.85–7.89 (m, 1H, Ar-H), 8.03 (d, *J* = 7.65 Hz, 1H, Ar-H), 8.61 (d, *J* = 8.5 Hz, 1H, Ar-H), 11.5 (s, 1H, N-H); ^13^C NMR(CDCl_3_, 213 MHz): δ 33.53, 39.17, 52.45, 110.02, 115.51, 120.53, 120.56, 120.92, 123.40, 124.23, 127.29, 127.65, 127.87, 129.58, 130.90, 133.96, 134.92, 135.05, 137.64, 140.47, 142.73, 149.60, 162.11, 164.49, 168.50. ESI MS: 568.67 [M+H]^+^, 570.67 [M+H+2]^+^; C_26_H_19_Cl_2_N_5_O_4_S (Calcd): C, 54.94; H, 3.37; N, 12.32; S, 5.64. Obsd: C, 54.87; H, 3.42; N, 12.39; S, 5.60.

### 3.2. Biological Activity

#### 3.2.1. Cytotoxicity

The cytotoxicity was assessed using the MTT method, according to our previously reported work [32]. The cell lines were purchased from American Type Culture Collection (ATCC). Details regarding the procedure are provided in the Supplementary Material.

#### 3.2.2. In Vitro EGFR Activity

EGFR inhibitory activity was assayed according to the reported method [44]. Erlotinib was used as a positive control. 

#### 3.2.3. Cell Cycle Analysis

The analysis was performed according to our previously published work [33]. Details regarding the procedure are provided in the Supplementary Material.

#### 3.2.4. Apoptosis Analysis

The assessment of apoptosis was performed using the Annexin V-FITC/PI analysis Kit, Cell Signaling Technology (CST), as instructed by the manufacturer [37]. 

#### 3.2.5. Statistical Analysis

Data are presented as mean ± SD of three different experiments, unless otherwise indicated. One-way ANOVA was used to test statistical significance (*p < 0.05, ** *p* < 0.01). 

### 3.3. Molecular Docking Studies

Glide-tools was used to achieve the molecular docking. The 3D crystal-structure for EGFR kinase was prepared using the Glide-tool, as described in [45]. All docking steps were carried out by ordinary methods of Maestro. 

## 4. Conclusions

The present study describes the benzimidazole-based 1,3,4-oxadiazole derivatives linked to 1,2,3-triazole/thioacetamide moieties as promising new EGFR inhibitors. Among all the tested compounds, two compounds, **10** and **13,** were found to be the most potent EGFR inhibitors, with IC_50_ 0.33 and 0.38 μM, respectively, and caused suppression of the cell cycle and induction of apoptosis at different phases in all the three tested cell lines, and these results were further supported by docking studies. These compounds possess potential as EGFR inhibitors in cancer treatment. 

## Figures and Tables

**Table 1:** In vitro antiproliferative activity of final compounds (**8–17**) against A549, SKOV3, and MDA-MB231 cells.

Compounds	IC_50_ (μM)		
	A549	SKOV3	MDA-MB231
8	26.65 ± 1.26	17.68 ± 0.68	15.49 ± 1.64
9	17.41 ± 0.54	13.60 ± 0.71	10.88 ± 0.58
**10**	**3.31 ± 0.14**	**6.98 ± 0.28**	**1.18 ± 0.68**
11	43.80 ± 0.87	25.69 ± 1.16	36.74 ± 0.75
12	37.15 ± 0.67	33.60 ± 0.91	25.18 ± 1.82
**13**	**5.30 ± 0.44**	**4.35 ± 0.38**	**2.90 ± 0.53**
14	11.64 ± 0.29	12.84 ± 0.53	20.85 ± 0.24
15	20.59 ± 1.41	13.71 ± 0.45	16.38 ± 0.44
16	7.83 ± 0.35	8.13 ± 0.28	10.49 ± 0.88
17	17.97 ± 0.87	12.18 ± 0.35	11.87 ± 0.91
doxorubicin	5.85 ± 0.61	8.65 ± 0.37	4.76 ± 0.44

IC_50_ values are the mean ± SD of three separate experiments.

**Table 2:** In vitro EGFR inhibitory activity of the active compounds.

Compounds	IC_50_ (µM)
9	0.95 ± 0.023
**10**	**0.33 ± 0.051**
**13**	**0.38 ± 0.022**
14	1.21 ± 0.047
16	1.09 ± 0.011
17	1.54 ± 0.026
Erlotinib	0.39 ± 0.034

IC_50_ values are the mean ± SD of three separate experiments; erlotinib: positive control.

**Table 3:** Binding energetic score for docking analysis for the final compounds against EGFR kinase (1M17).

Compd	Δ_Ε_	RMSD	E_conf_	E_place_	E-_Int._	LE	Ki	Fit Quality
8	**−8.01**	2.05	−7.45	−19.93	−9.63	−4.42	1.49	−1.80
9	**−8.16**	2.28	53.09	−29.59	−9.09	−4.67	1.54	−1.63
10	**−8.27**	1.30	19.01	−26.40	−9.21	−6.93	1.94	−3.91
11	−7.33	2.79	57.17	−24.50	−9.22	−8.65	2.16	−5.91
12	−7.83	1.61	34.88	−24.49	−10.29	−5.66	1.73	−2.82
13	**−8.31**	1.34	23.09	−16.95	−10.68	−6.14	1.81	−3.14
14	−7.23	1.99	39.33	−26.71	−9.58	−7.24	1.98	−4.59
15	−7.25	4.15	−3.82	−24.83	−10.81	−1.17	0.16	1.96
16	−6.06	3.36	−11.40	−30.67	−9.46	−4.77	1.56	−1.79
17	−7.87	1.16	41.09	−41.70	−2.56	−2.56	0.94	0.55
Erlotinib	−7.90	1.94	−30.17	−15.64	−14.74	−8.39	2.47	0.11

Δ_E_, E_conf_ and E_place_ are the free binding energy for the ligand from a given pose, conformer, and receptor; E-_Int._: the ligand’s affinity binding energy with the receptor; RMSD: root mean square deviation between the docking pose and the co-crystal ligand.

## Data Availability

Not applicable.

## Supplementary Materials

The following supporting information can be downloaded at: https://www.mdpi.com/article/10.3390/molecules27206899/s1, Figure S1–S30: NMR (^1^H & ^13^C) and mass spectra of final compounds; Table S1: In silico toxicity prediction of compounds **10** and **13**, detailed procedure for anticancer activity.
