# Supplementary material for: Cell Cycle Arrest and Apoptosis-Inducing Ability of Benzimidazole Derivatives: Design, Synthesis, Docking, and Biological Evaluation
Source: Molecules. 2022 Oct 14;27(20):6899. doi: 10.3390/molecules27206899 (PMC9607330; doi:10.3390/molecules27206899)

SUPPLEMENTARY MATERIAL

Cell cycle arrests and apoptosis inducing ability of benzimidazole derivatives: Design, synthesis, docking and biological evaluation

Syed Nazreen[1*], Abdulraheem SA Almalki[2], Serag Eldin I. Elbehairi[3,4], Ali A. Shati[3], Mohammad Y. Alfaifi[3], Ahmed A. Elhenawy[1,5], Nawaf I. Alsenani[1], Anas Alfarsi[1], Abdulrahman Alhadhrami[2], Esam A. Alqurashi[1], Mohammad Mahboob Alam[1,*]

Experimental

Biological activity

Cytotoxicity

Roswell Park Memorial Institute Medium (RPMI 1640), Gibco, USA, was used to culture A549 (human lung adenocarcinoma), MDA MB 231 (breast adenocarcinoma) and SKOV3 (ovary adenocarcinoma) cell lines. FBS (10%) and 100 units/mL PS (penicillin/streptomycin) were supplied to that same media. The cells incubated at 37˚C in a humidified environment with 5% carbon dioxide. The activity was performed according to our reported work [32]. The positive control used was Tamoxifen and the O.D. was calculated at 540 nm using a microplate reader (FluoStar Omega (BMG Labtec, Ortenberg, Germany).

Cell cycle analysis

The most promising compounds (8, 9, 10, 13) with IC_{50}s values were pre-calculated and delivered to cancer cells for 48 hours. A549, MDA,MB 231 and SKOV3 cells were then trypsinized, washed twice in PBS (phosphate buffered saline), and then resuspended in propidium iodide (PI) (500 µL) with RNase staining buffer from Cell Signaling Technology (CST) and incubated for 15 minutes. Fluorescence activated cell sorting analysis were carried out using a Cytek® Northern Lights 2000 spectral flow cytometer using SpectroFlo™ Software version 2.2.0.3, to evaluate the data from 10,000 cells and the distribution of cell cycle phases for each sample [37].

Apoptosis analysis

LS174T cells treated with compounds (8, 9, 10, 13) for 48 hr. and then trypsinized and washed twice with PBS. The assessment of apoptosis was performed using Annexin V-FITC/PI analysis Kit, Cell Signaling Technology (CST), as instructed by the manufacturer [38].

Table S1: *In silico* toxicity study of compounds **10** and **13**

**Compounds**	**Max. Tolerated dose (Human) mg/kg/day**	**Oral rat acute toxicity (LD50) mol/kg**	**Oral rat chronic toxicity (LOAEL) mg/kg bw/day**	**Hepatotoxicity**	**Skin Sensitization**
10	0.3	2.438	-0.145	No	No
13	0.125	2.472	-0.344	No	No
Erlotinib	0.002	2.368	0.88	Yes	No

Figure Captions:

Figure S1-S10: ^{1}H NMR of final compounds 8-17

Figure S11-S20: ^{13}C NMR of final compounds 8-17

Figure S21-S30: Mass of final compounds 8-17

Figure S1: ^{1}H NMR of Compound 8

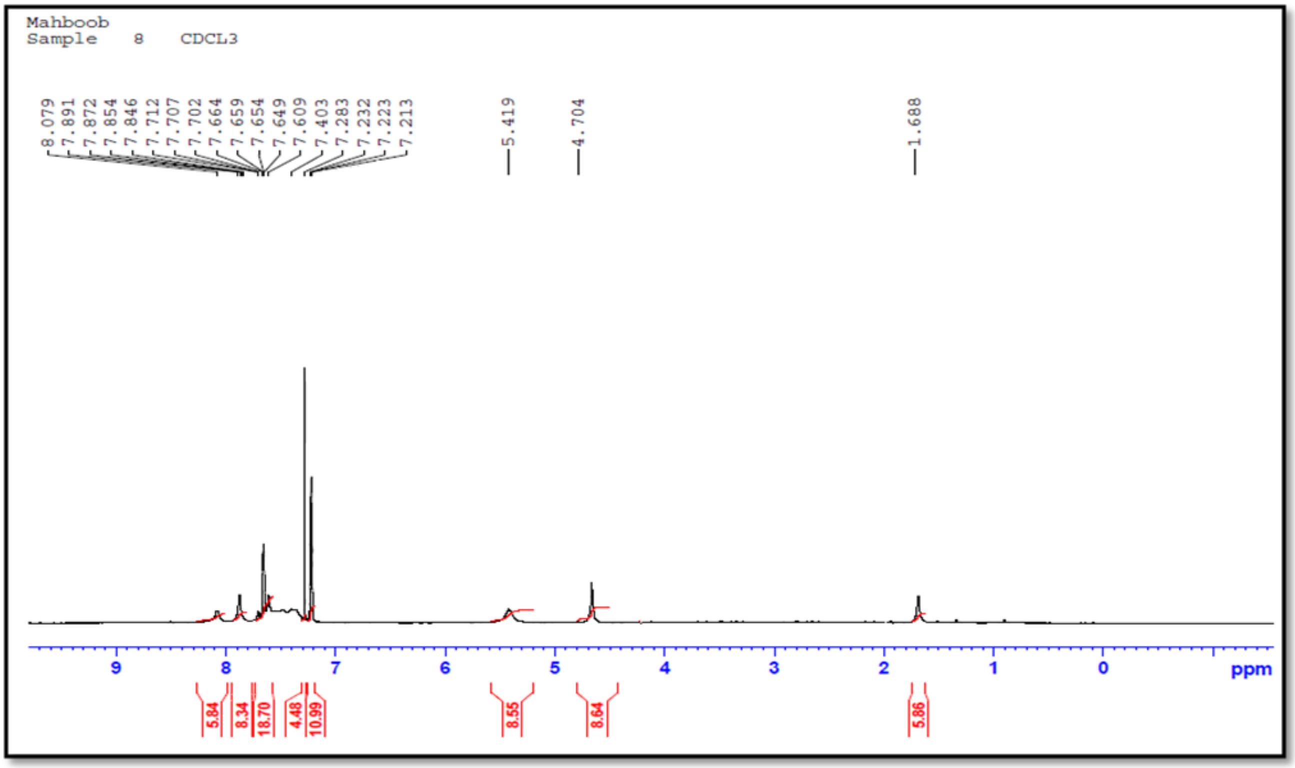

Figure S2: ^{1}H NMR of Compound 9

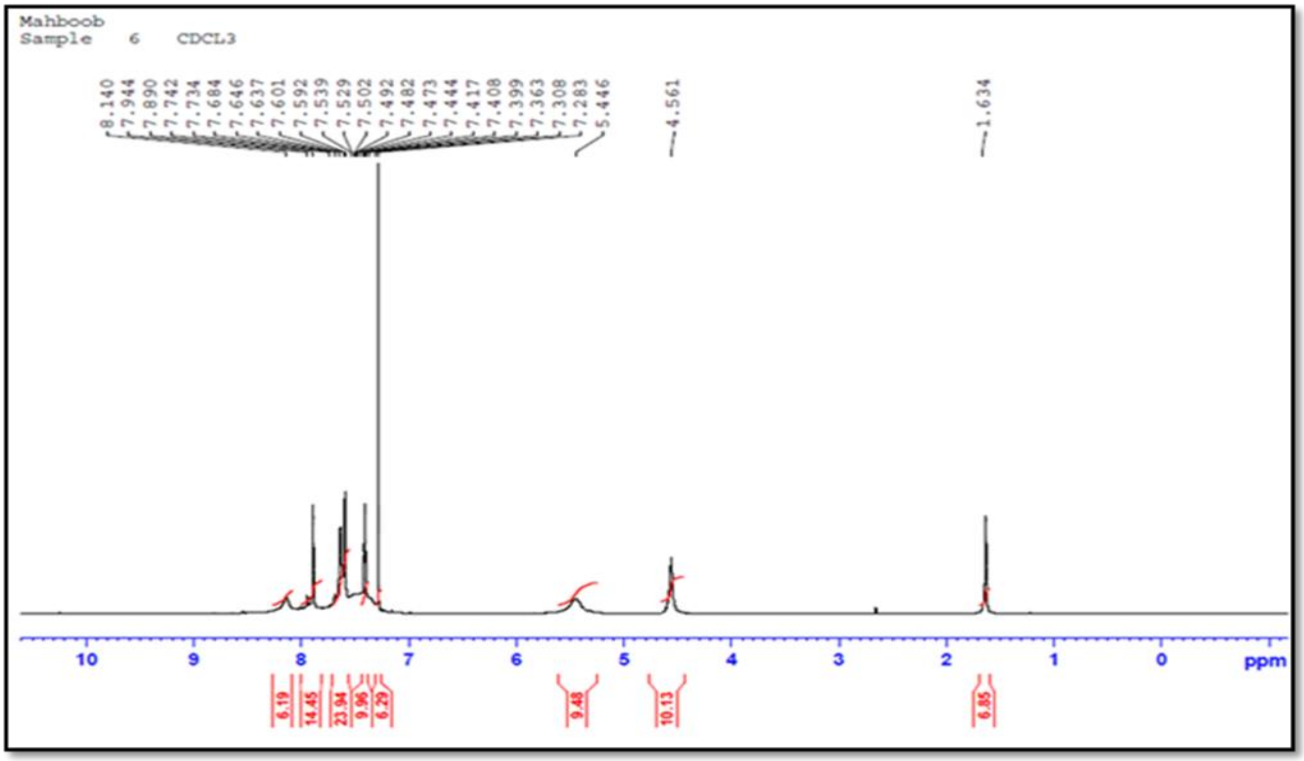

Figure S3: ^{1}H NMR of Compound 10

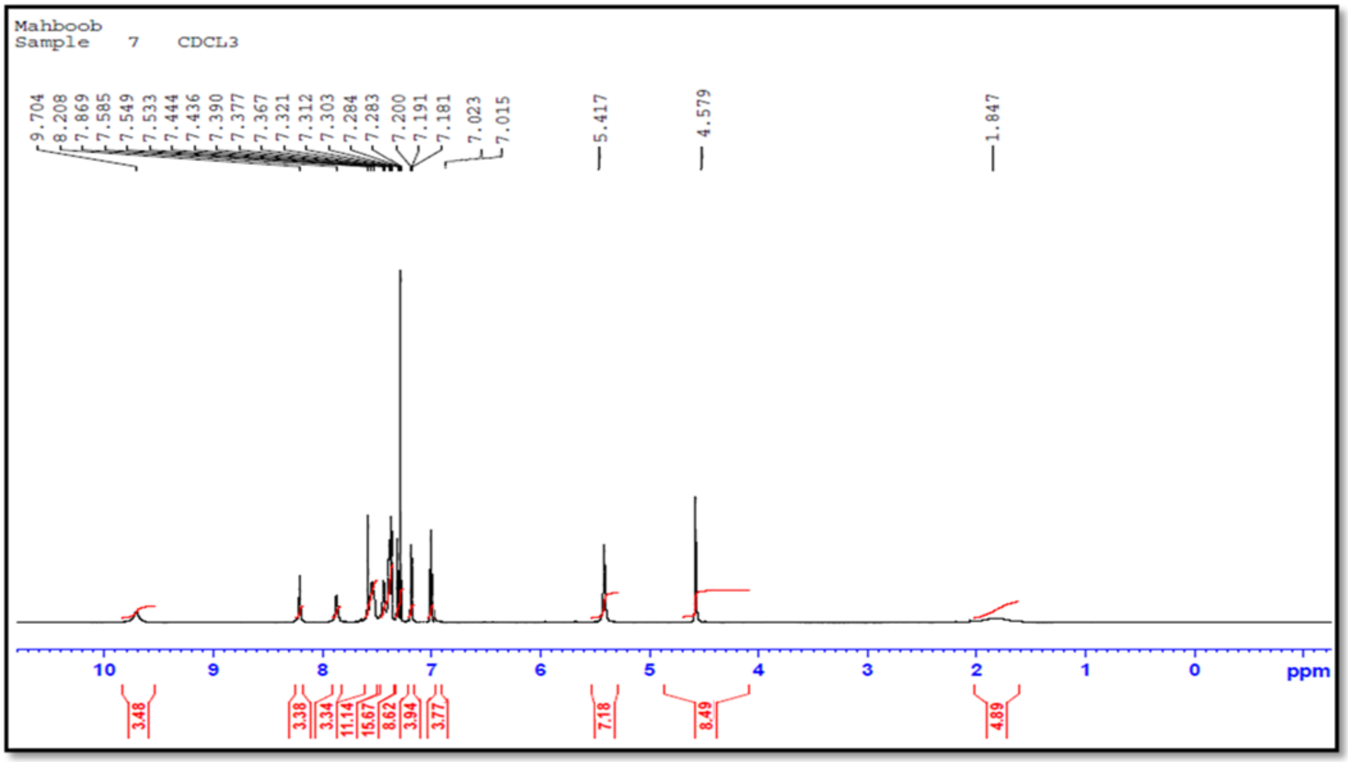

Figure S4: ^{1}H NMR of Compound 11

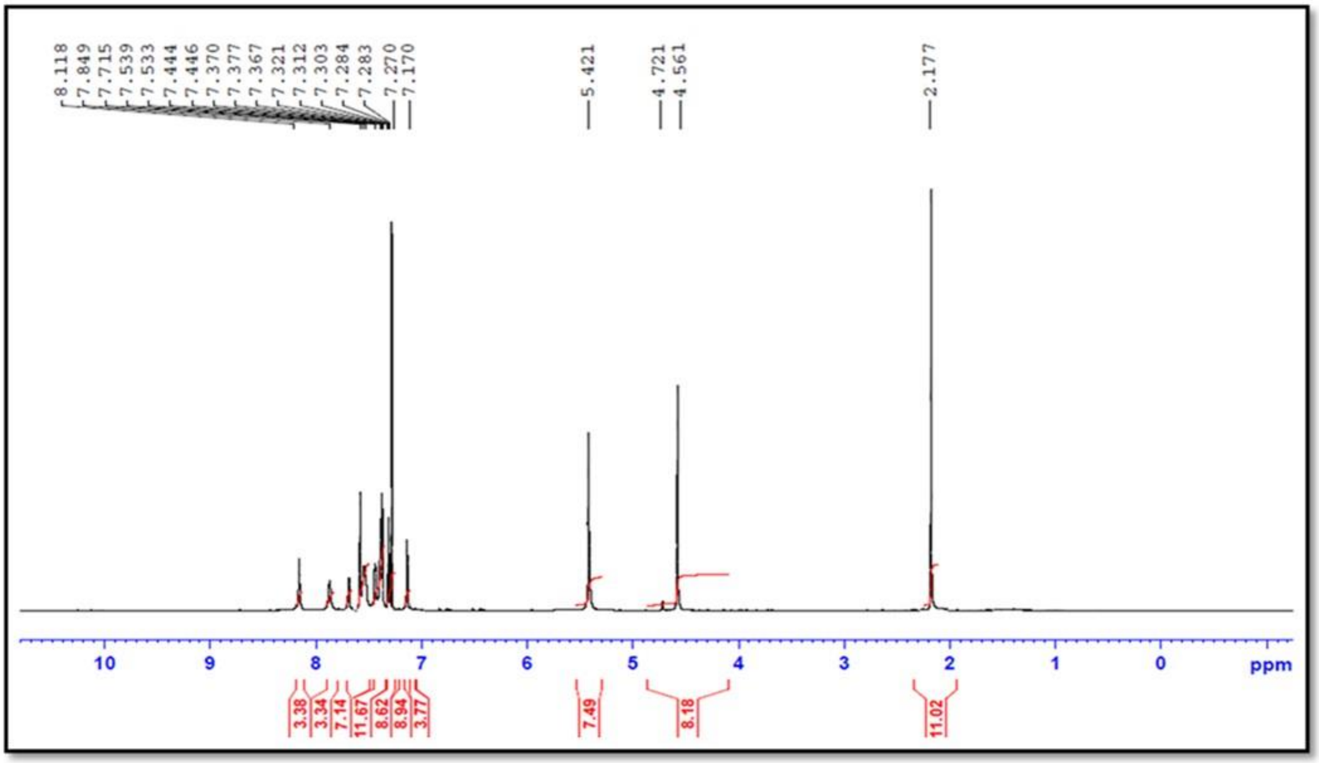

Figure S5: ^{1}H NMR of Compound 12

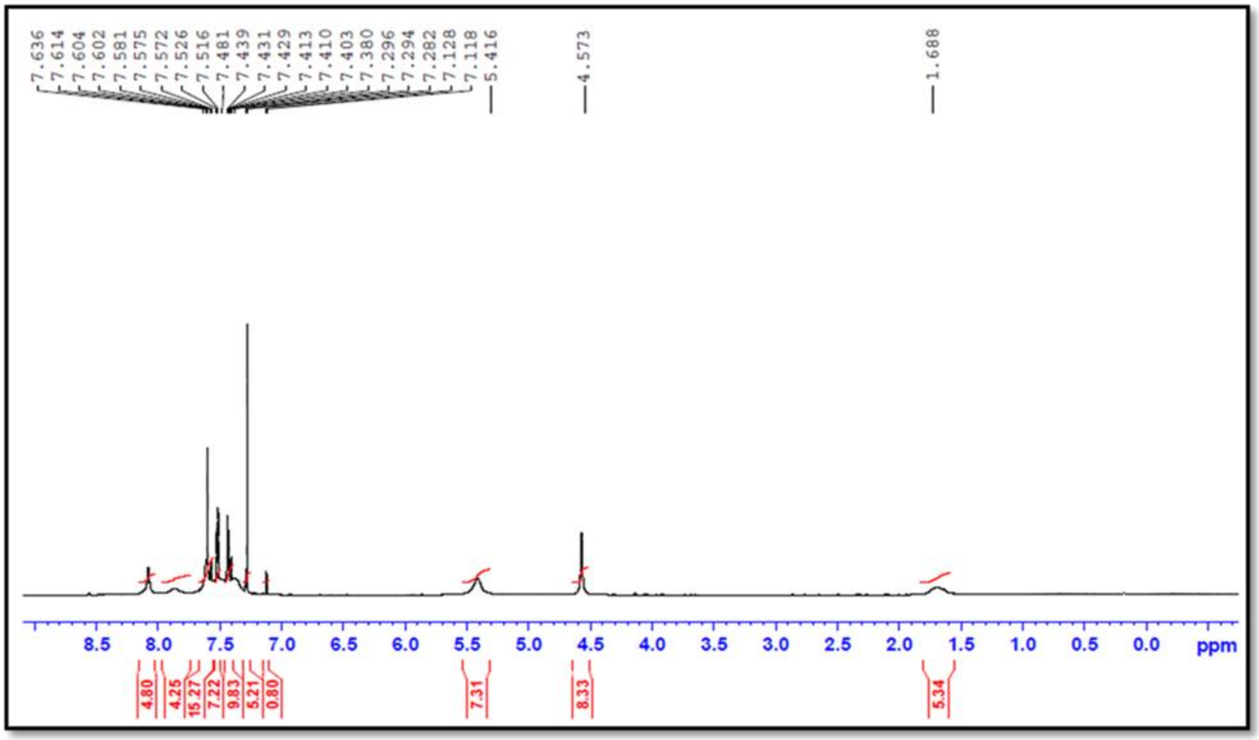

Figure S6: ^{1}H NMR of Compound 13

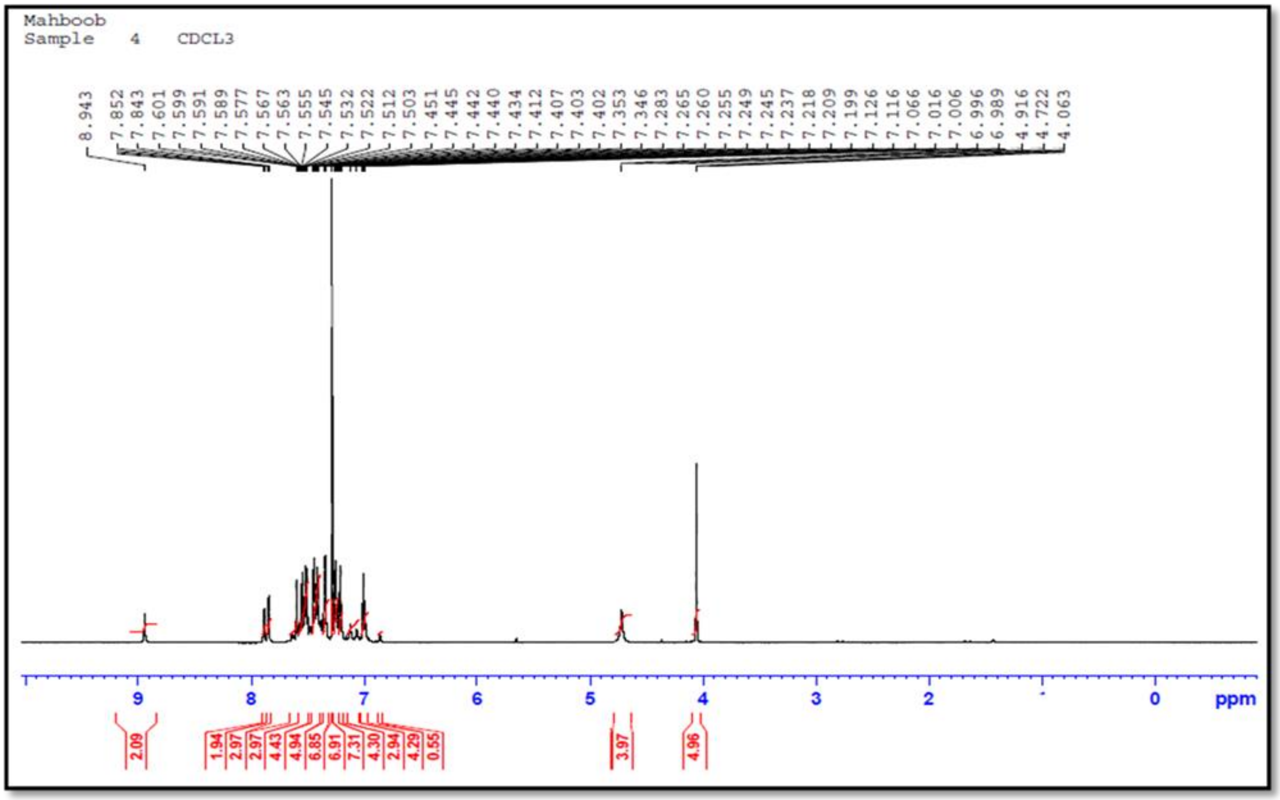

Figure S7: ^{1}H NMR of Compound 14

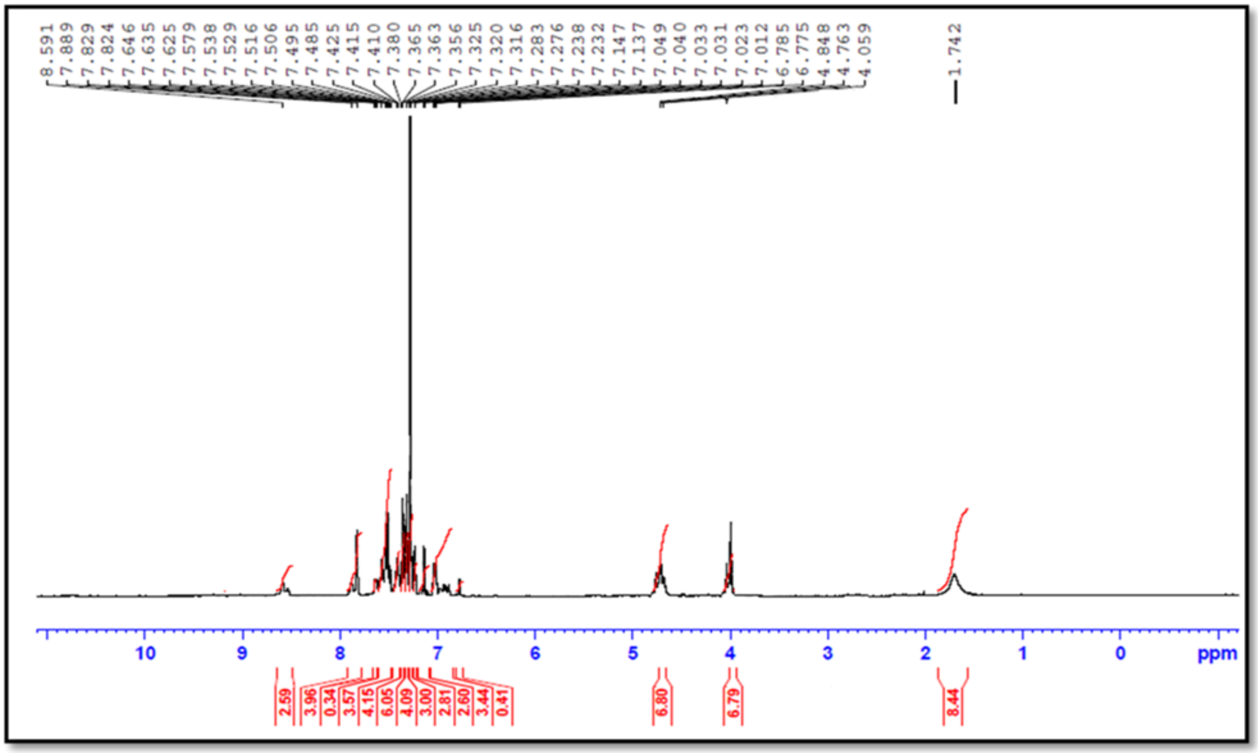

Figure S8: ^{1}H NMR of Compound 15

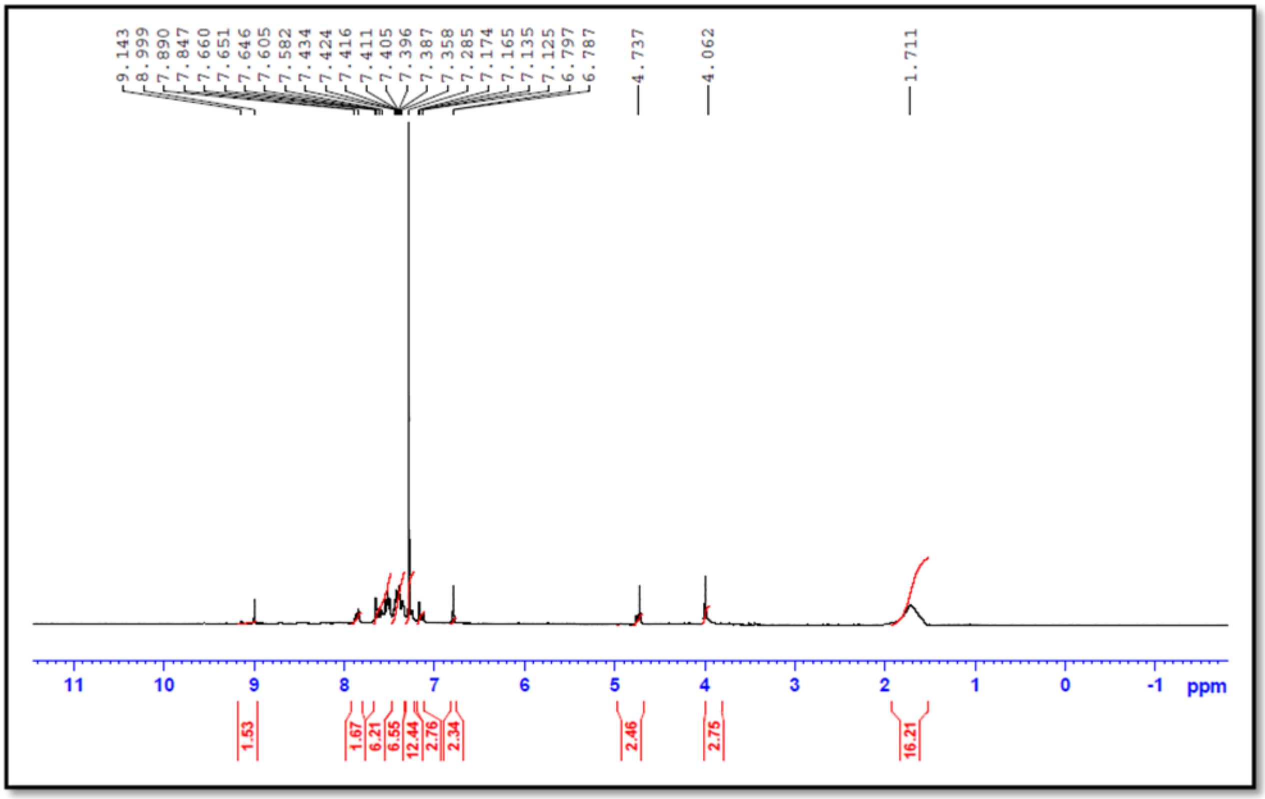

Figure S9: ^{1}H NMR of Compound 16

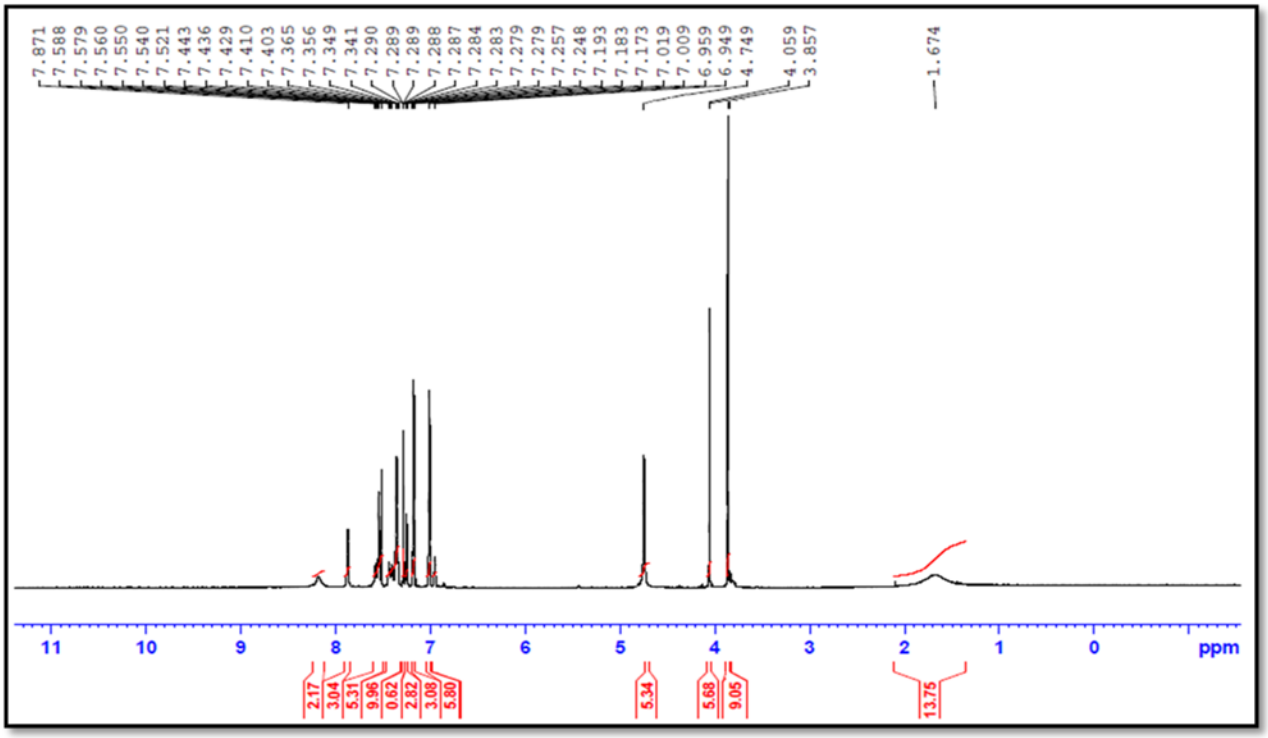

Figure S10: ^{1}H NMR of Compound 17

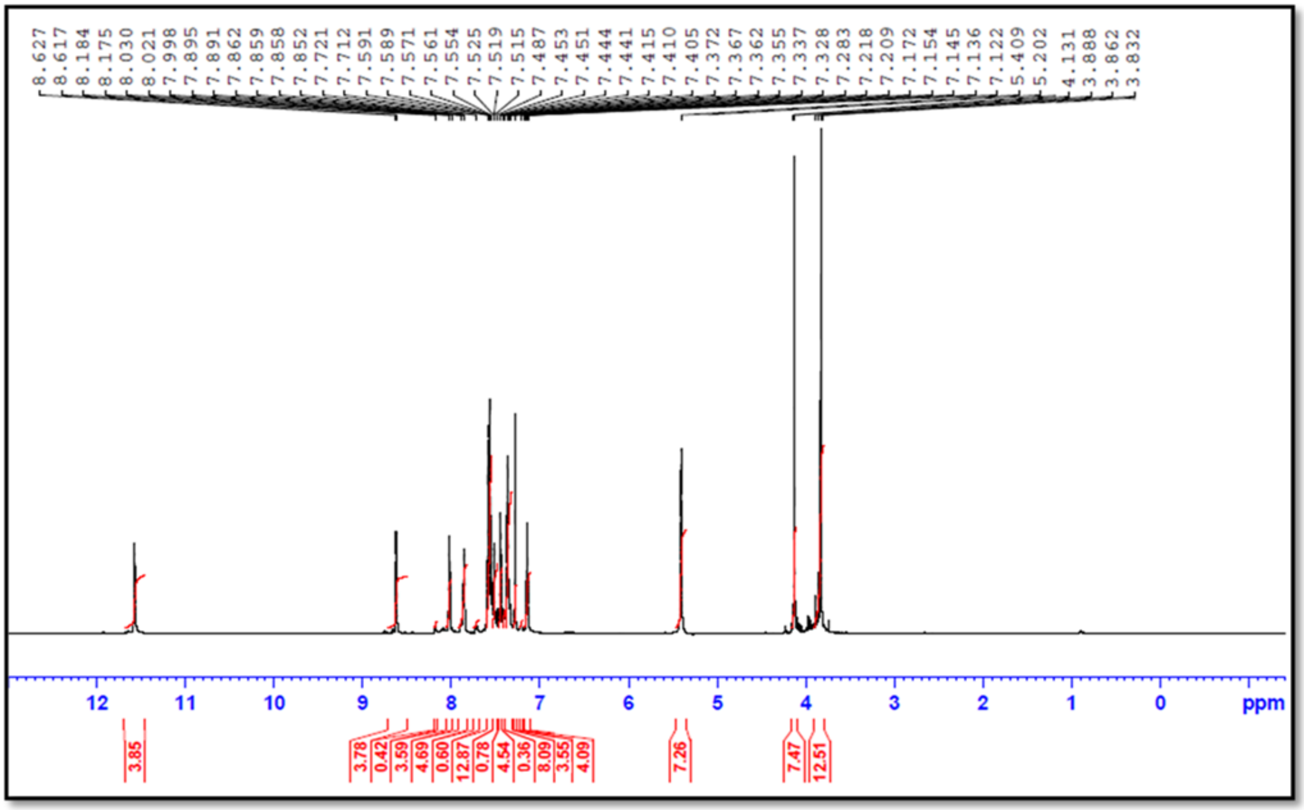

Figure S11: ^{13}C NMR of Compound 8

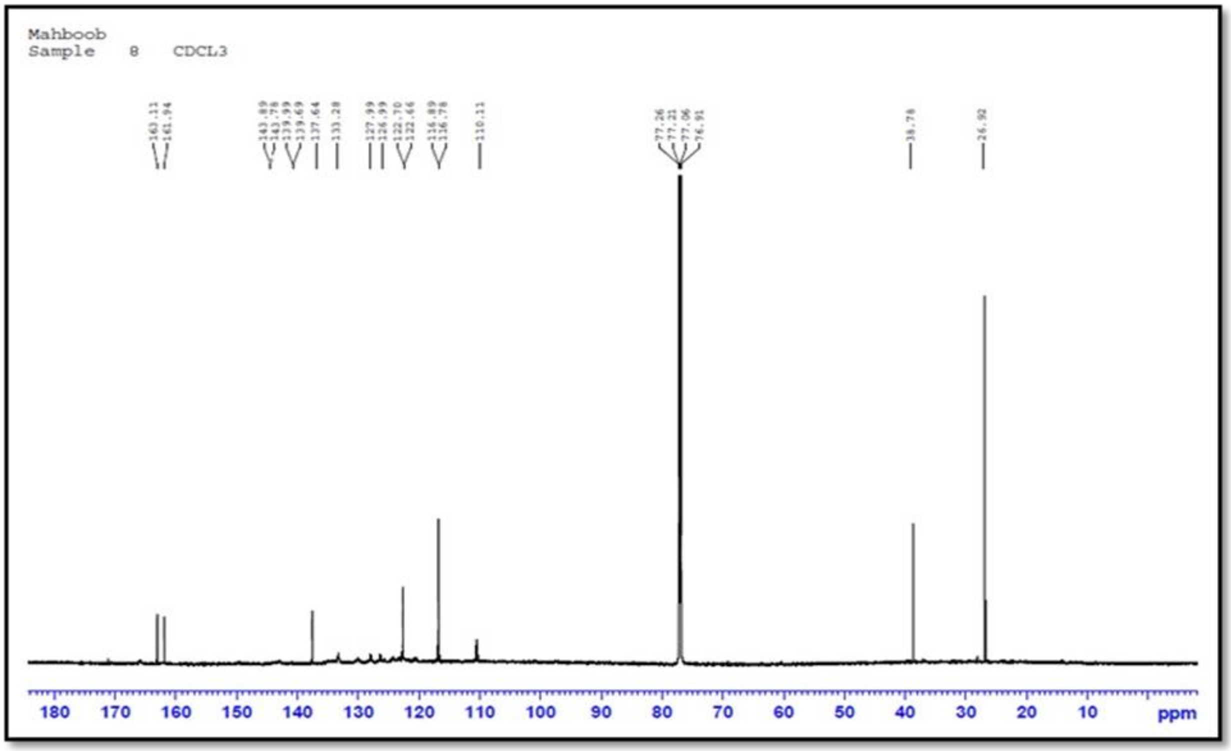

Figure S12: ^{13}C NMR of Compound 9

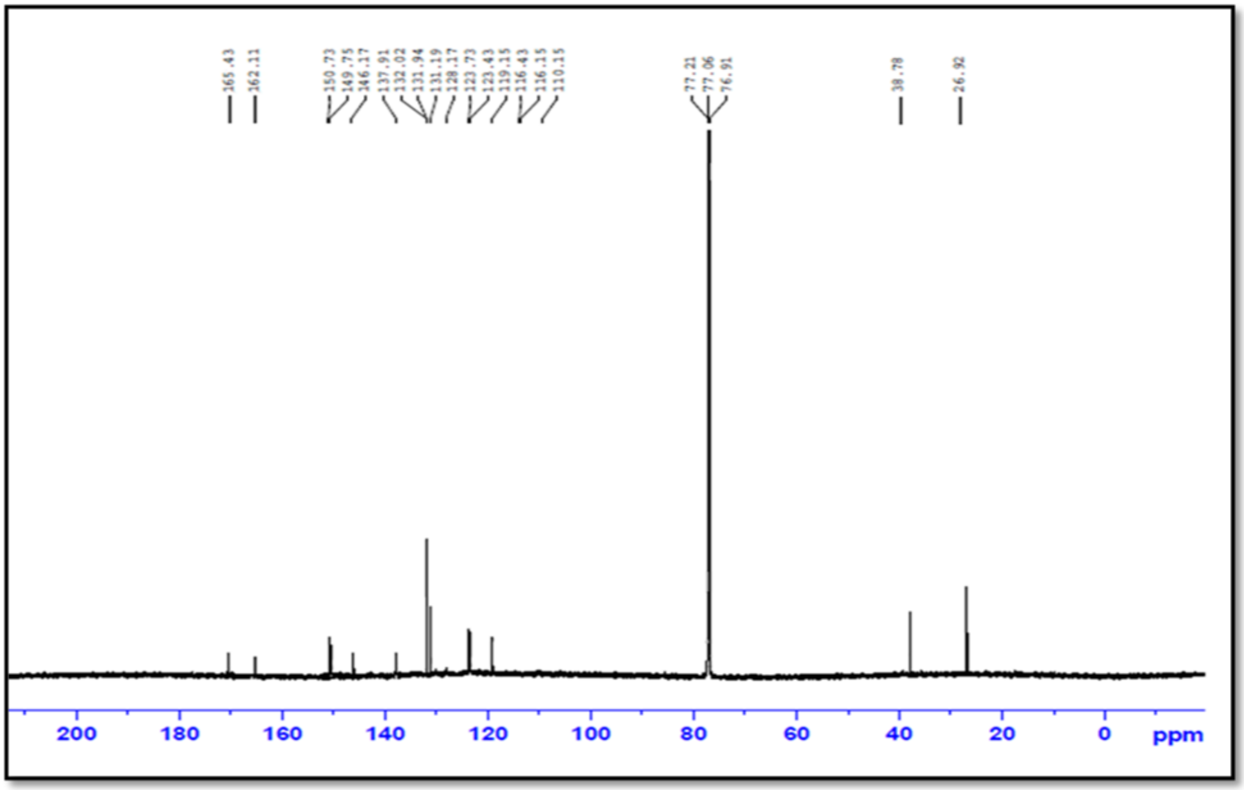

Figure S13: ^{13}C NMR of Compound 10

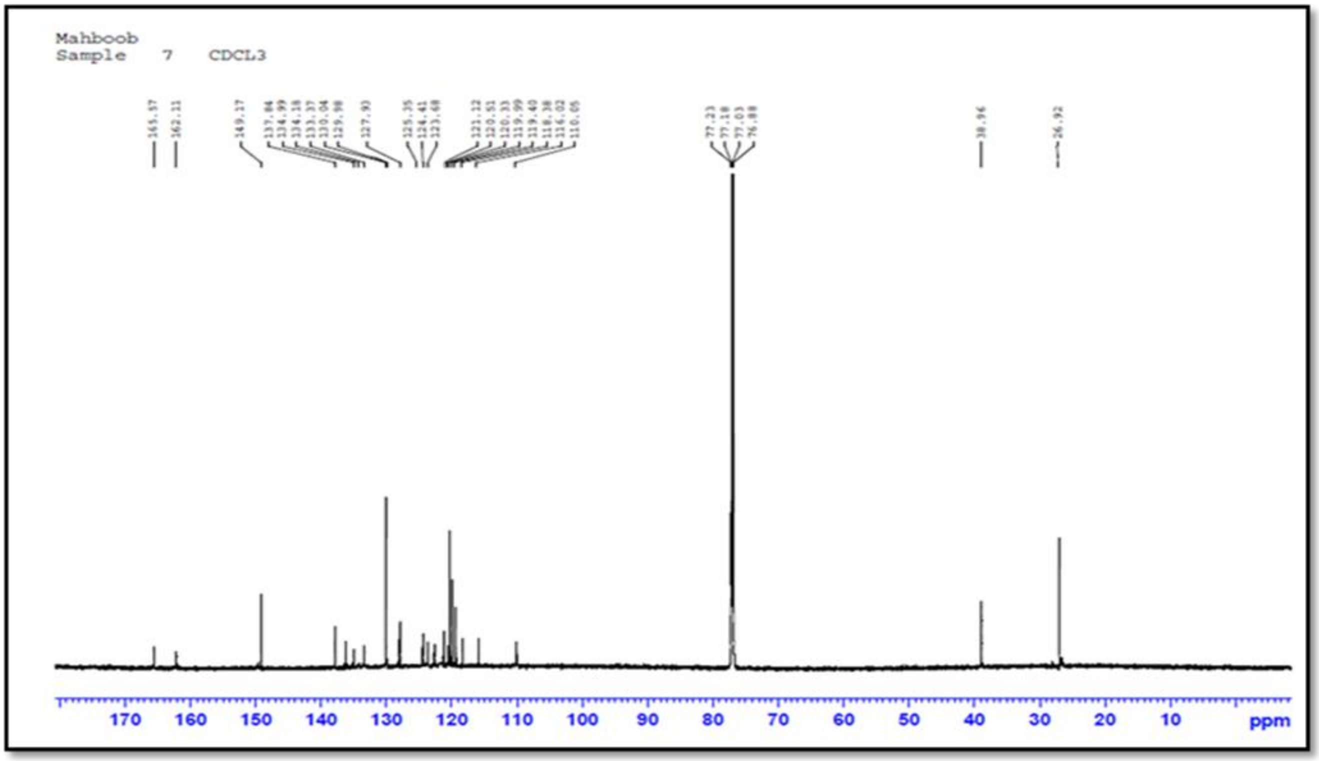

Figure S14: ^{13}C NMR of Compound 11

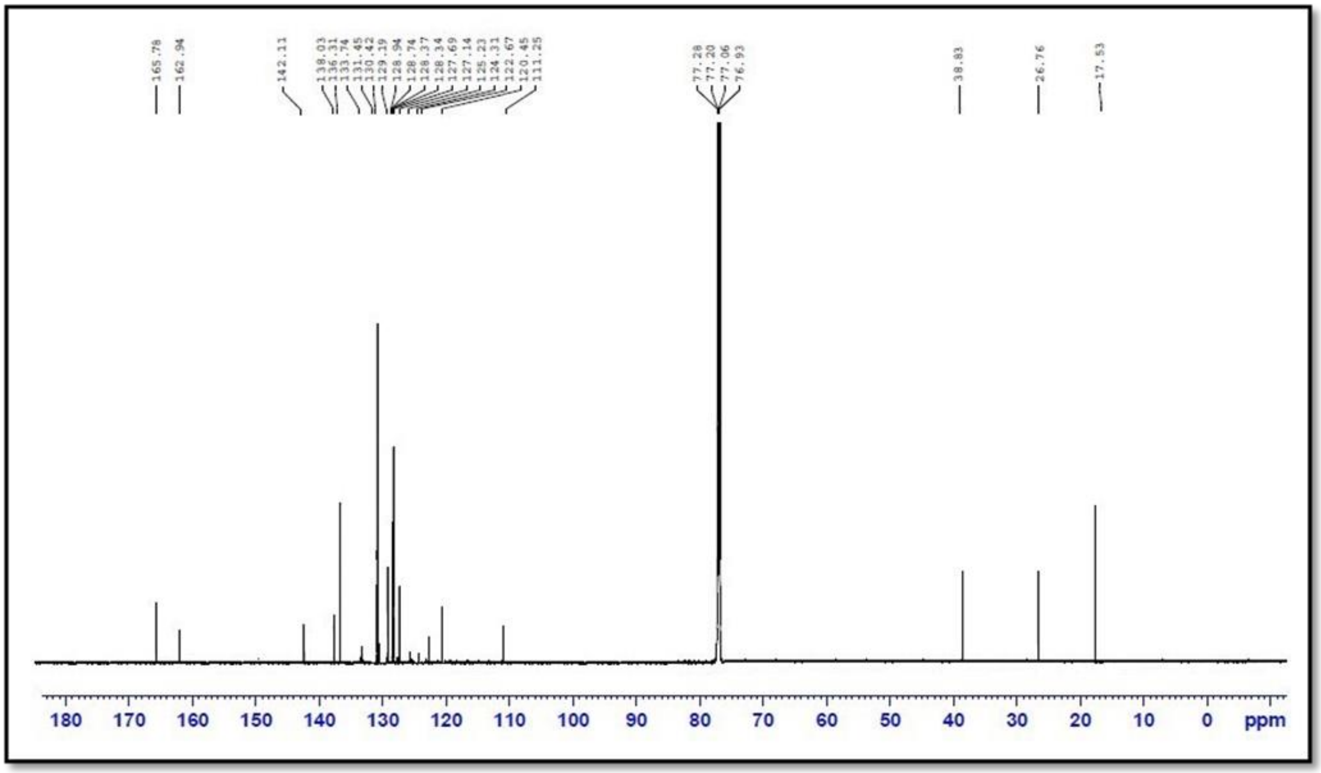

Figure S15: ^{13}C NMR of Compound 12

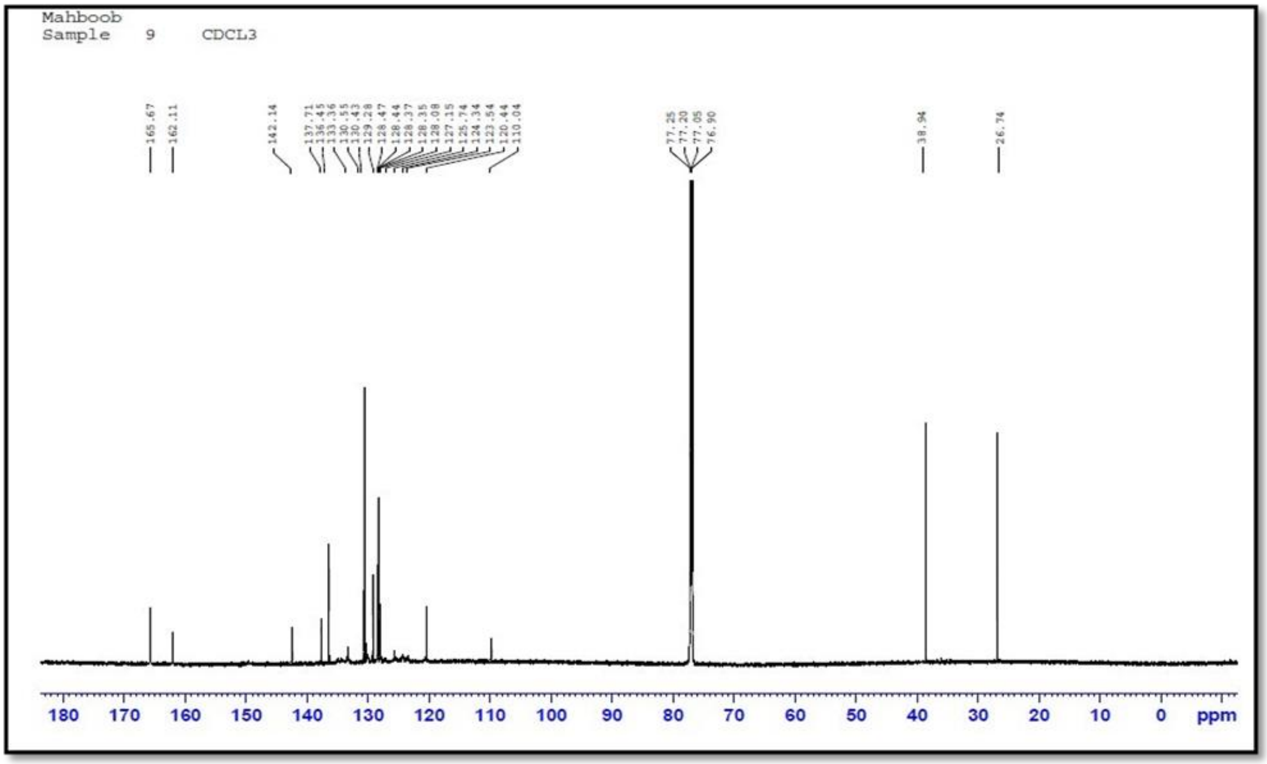

Figure S16: ^{13}C NMR of Compound 13

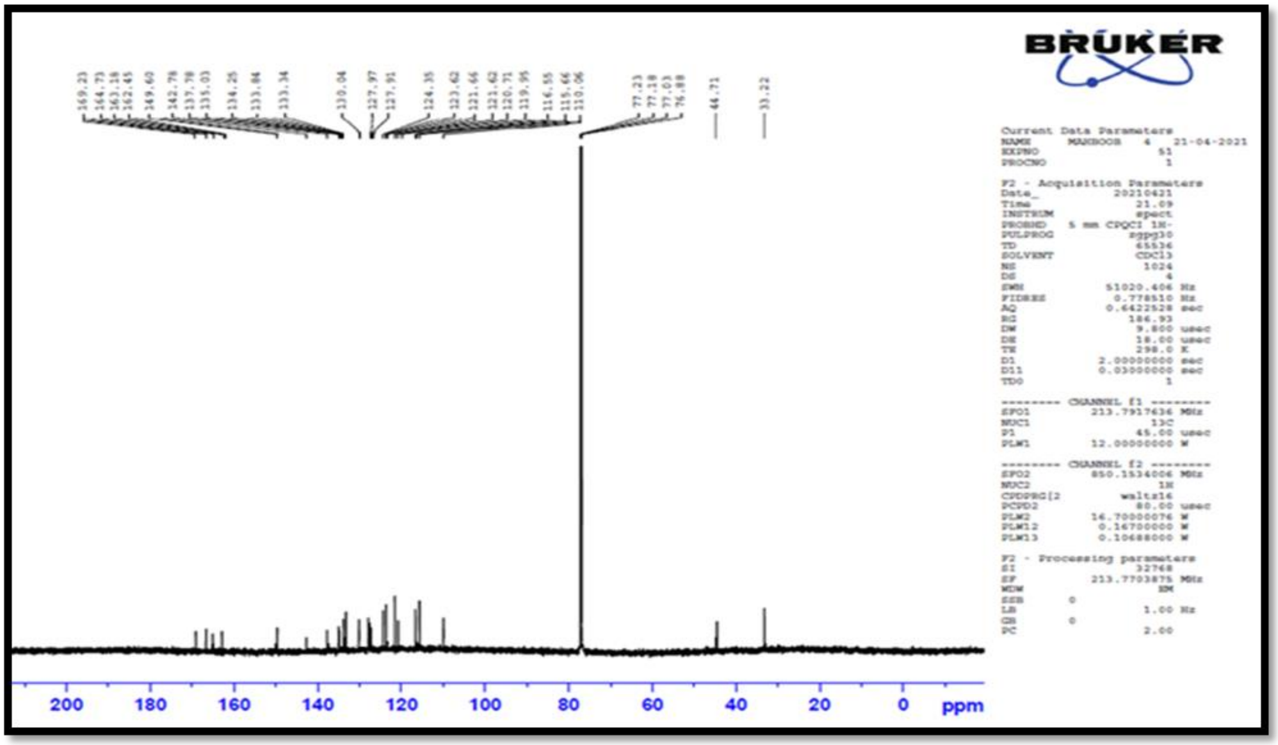

Figure S17: ^{13}C NMR of Compound 14

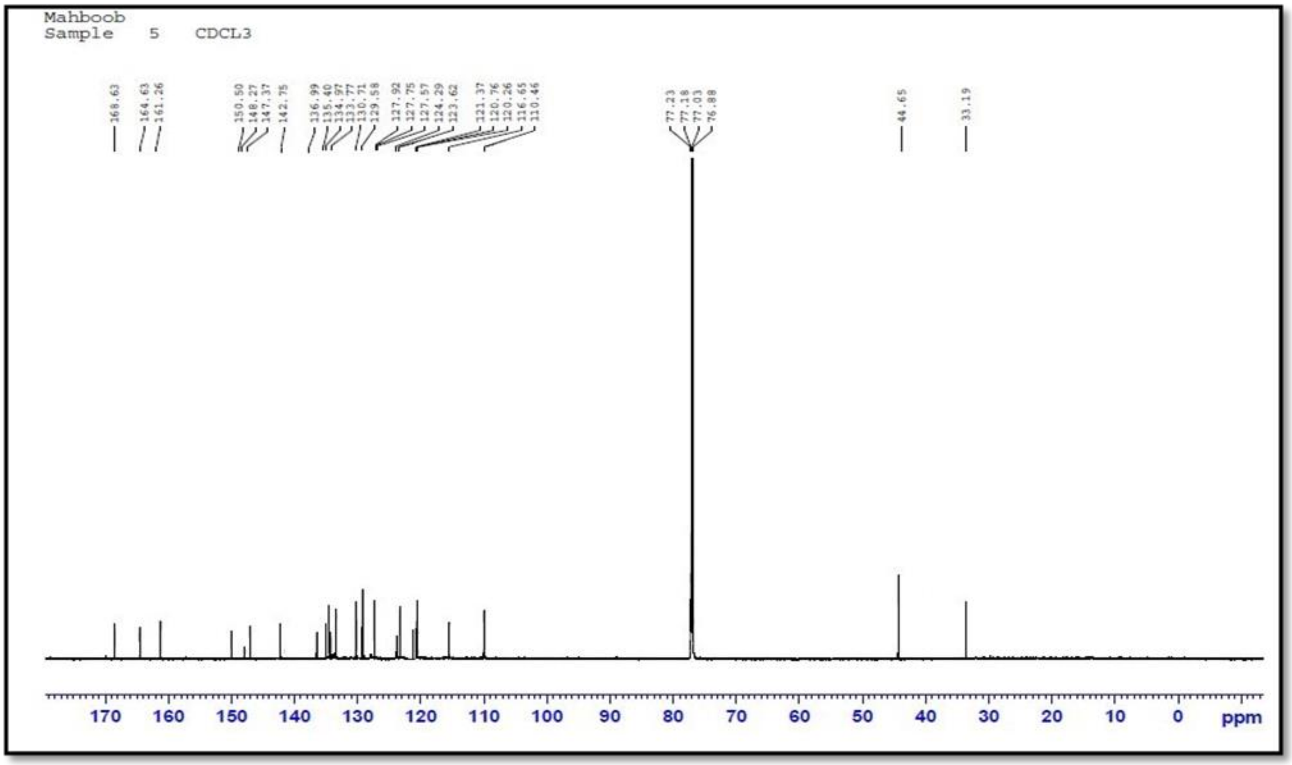

Figure S18: ^{13}C NMR of Compound 15

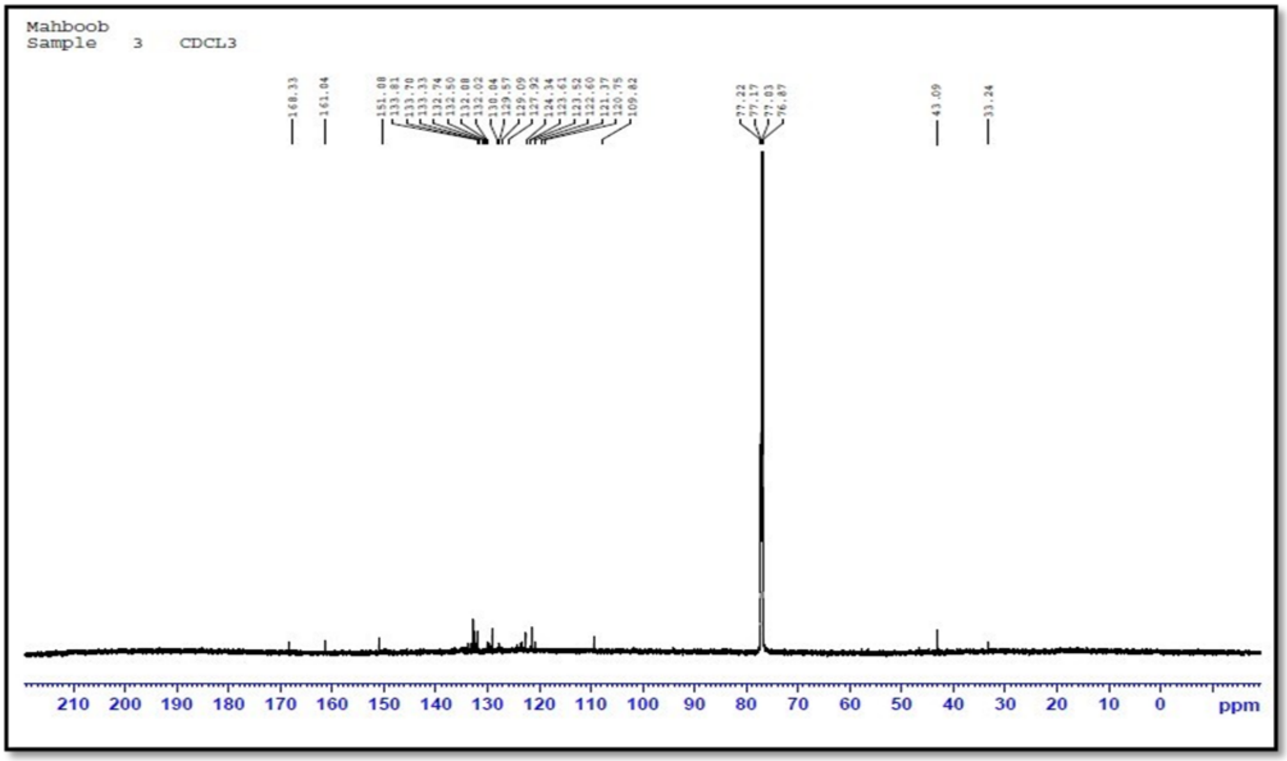

Figure S19: ^{13}C NMR of Compound 16

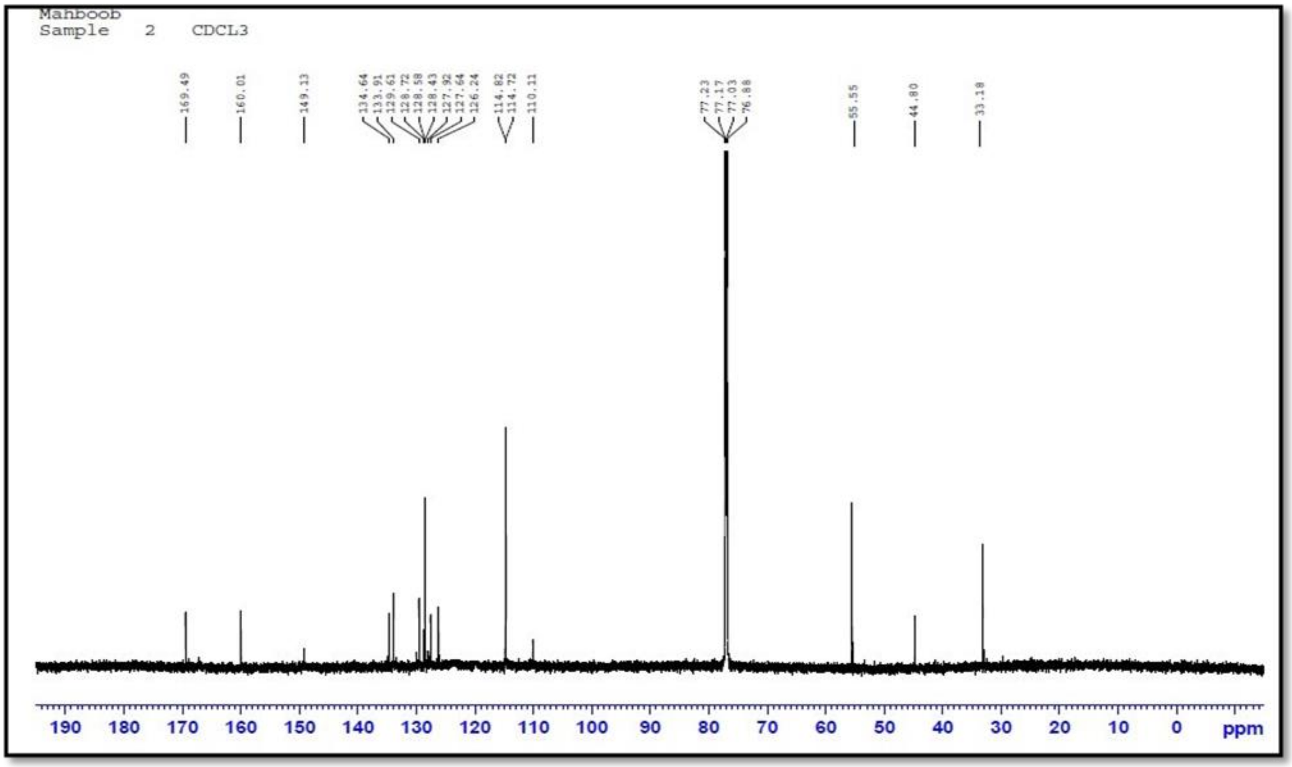

Figure S20: ^{13}C NMR of Compound 17

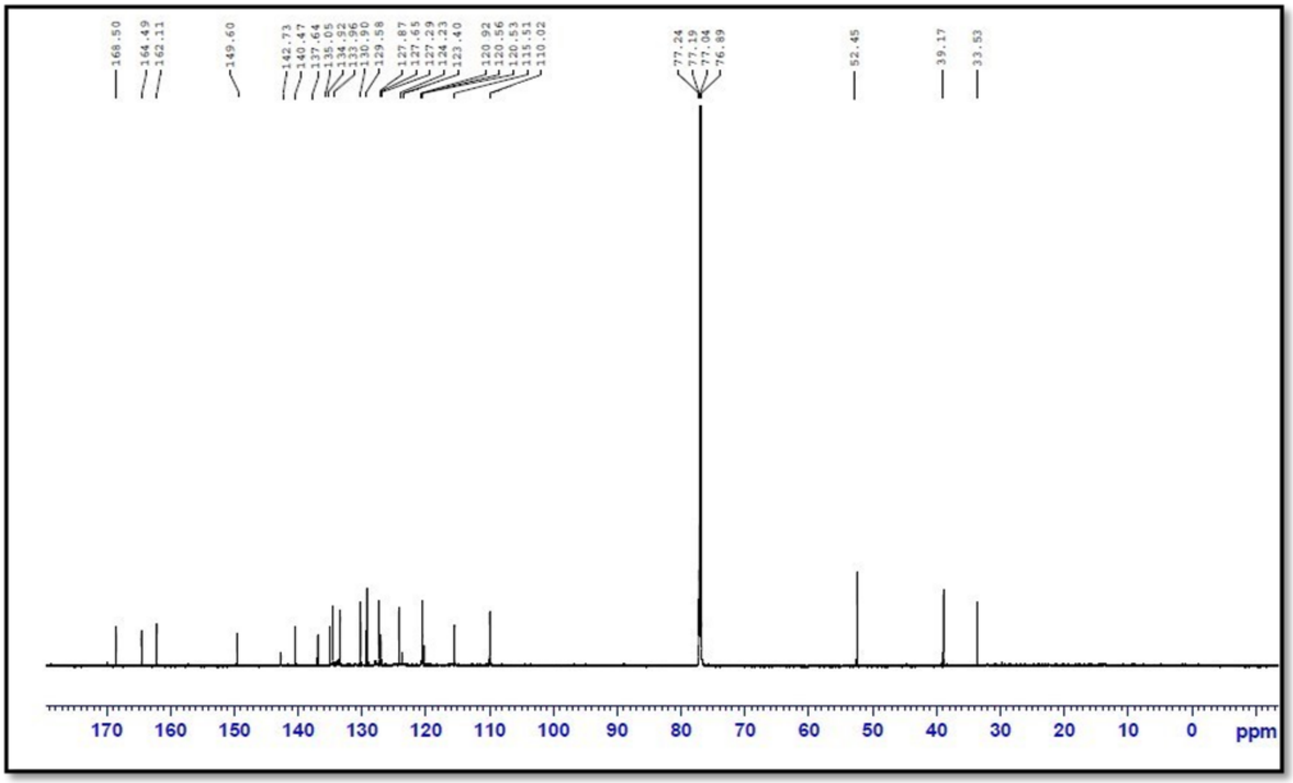

Figure S21: Mass of Compound 8

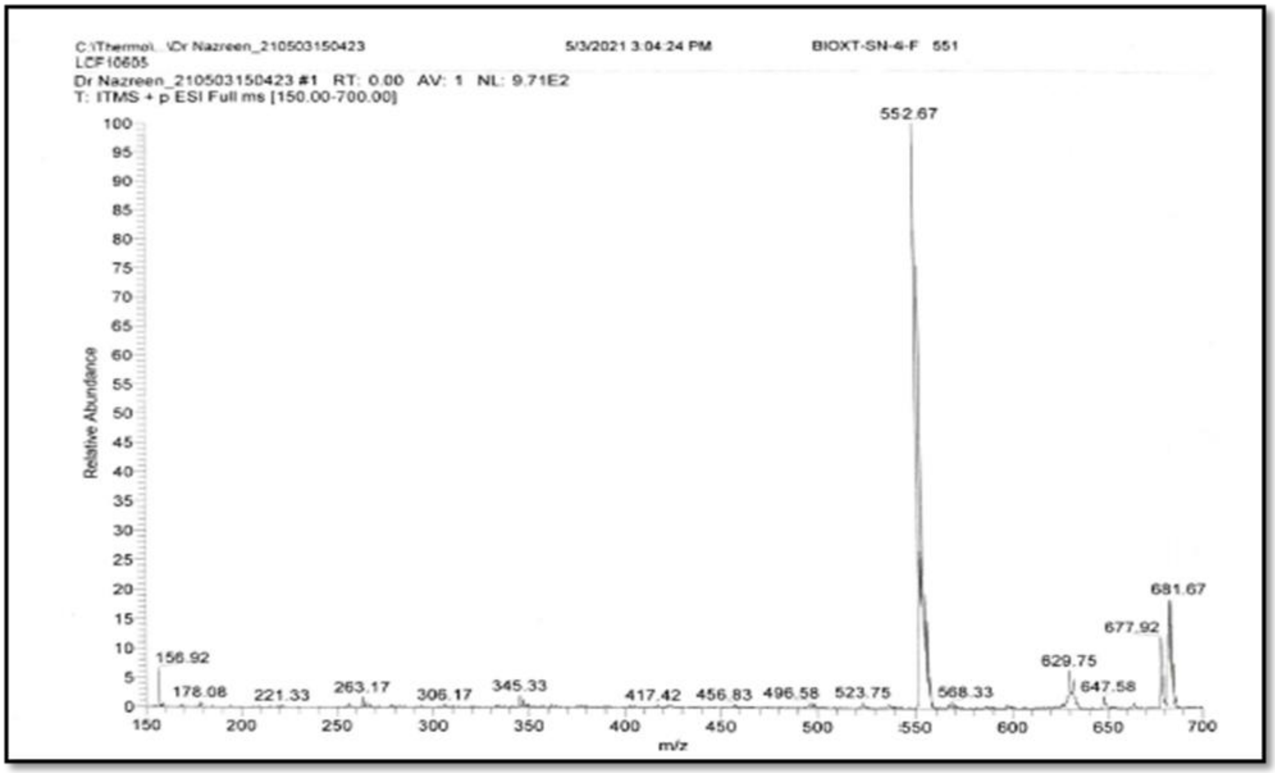

Figure S22: Mass of Compound 9

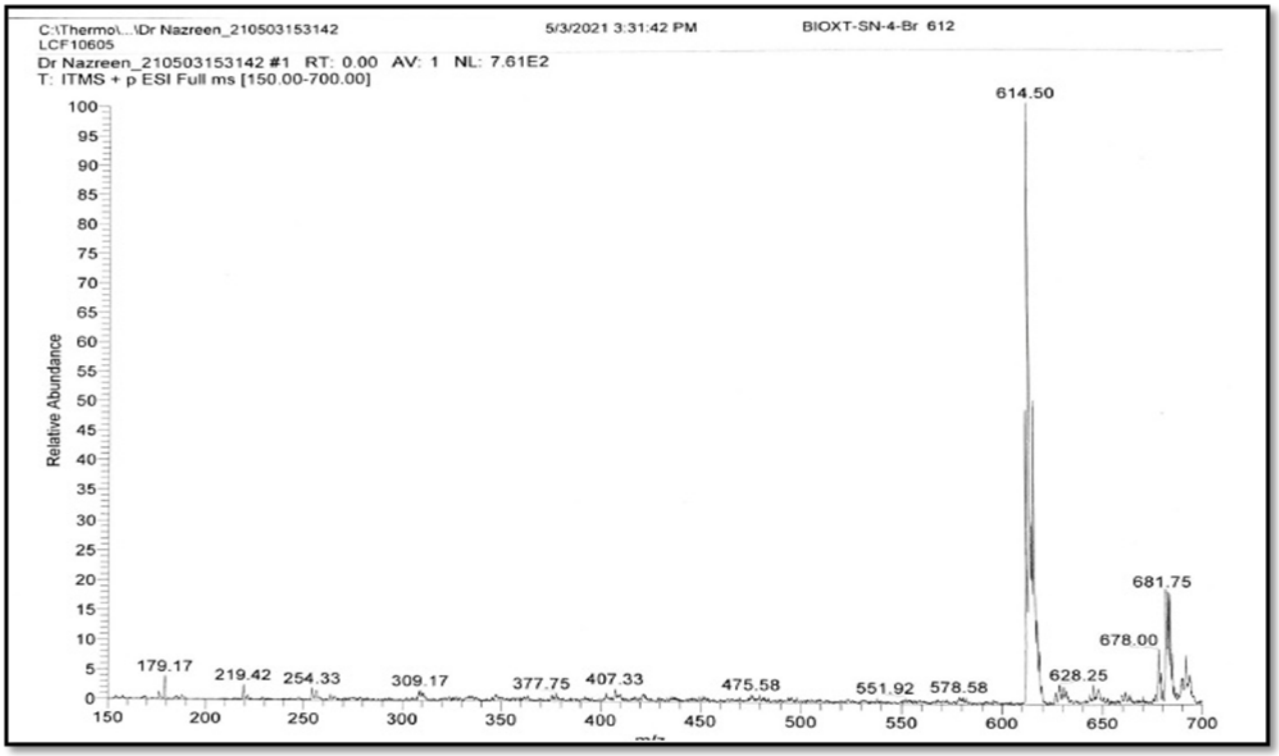

Figure S23: Mass of Compound 10

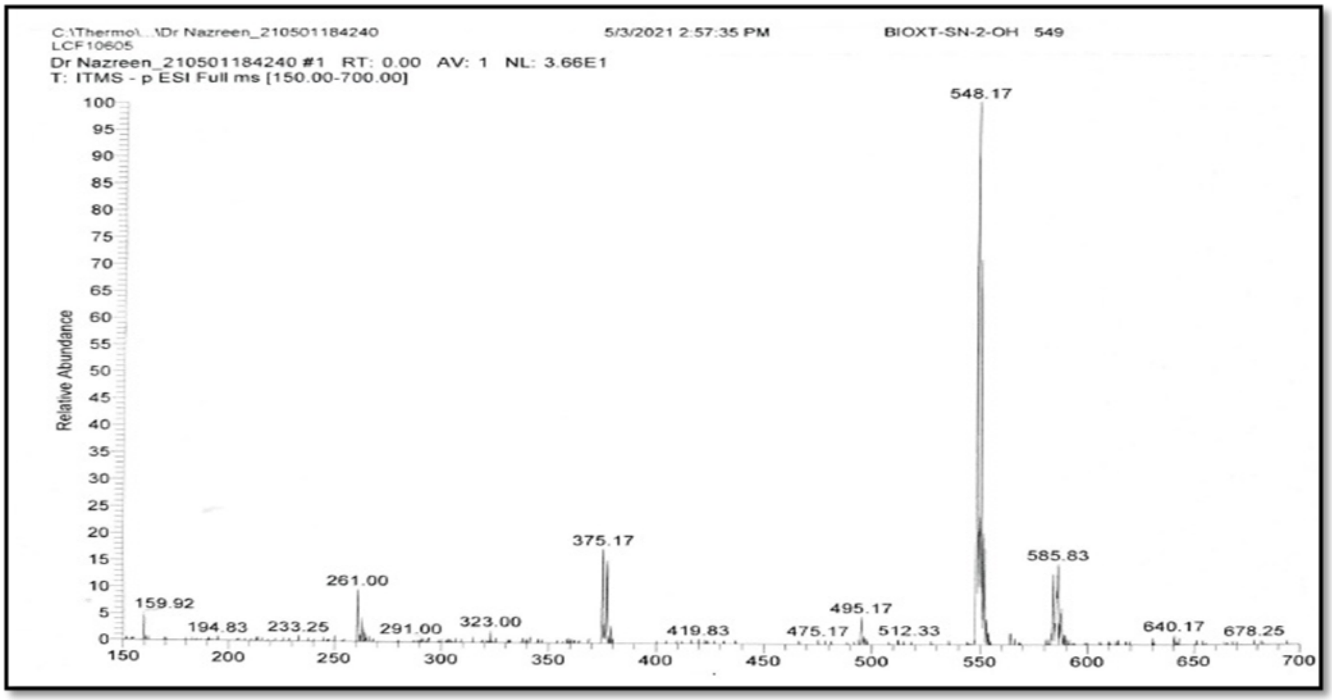

Figure S24: Mass of Compound 11

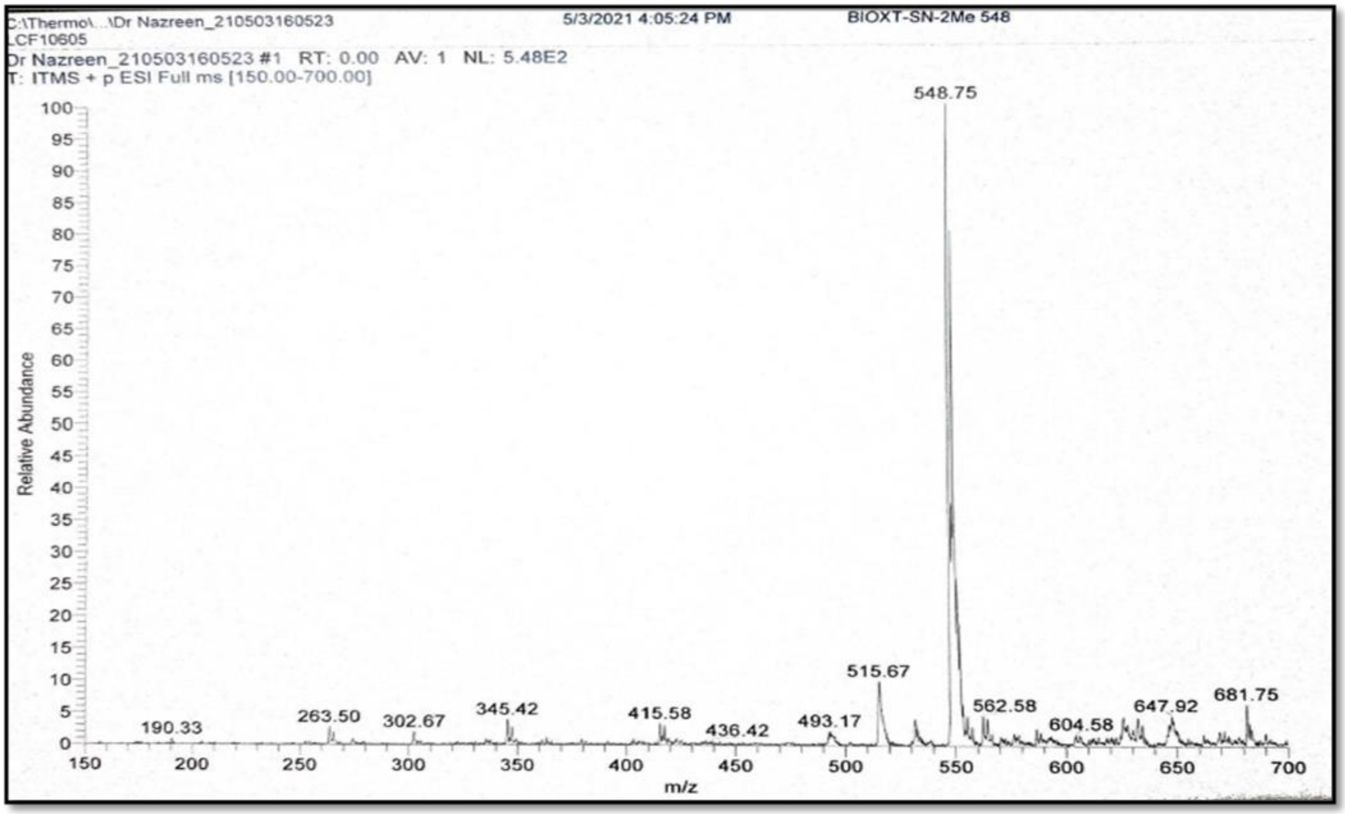

Figure S25: Mass of Compound 12

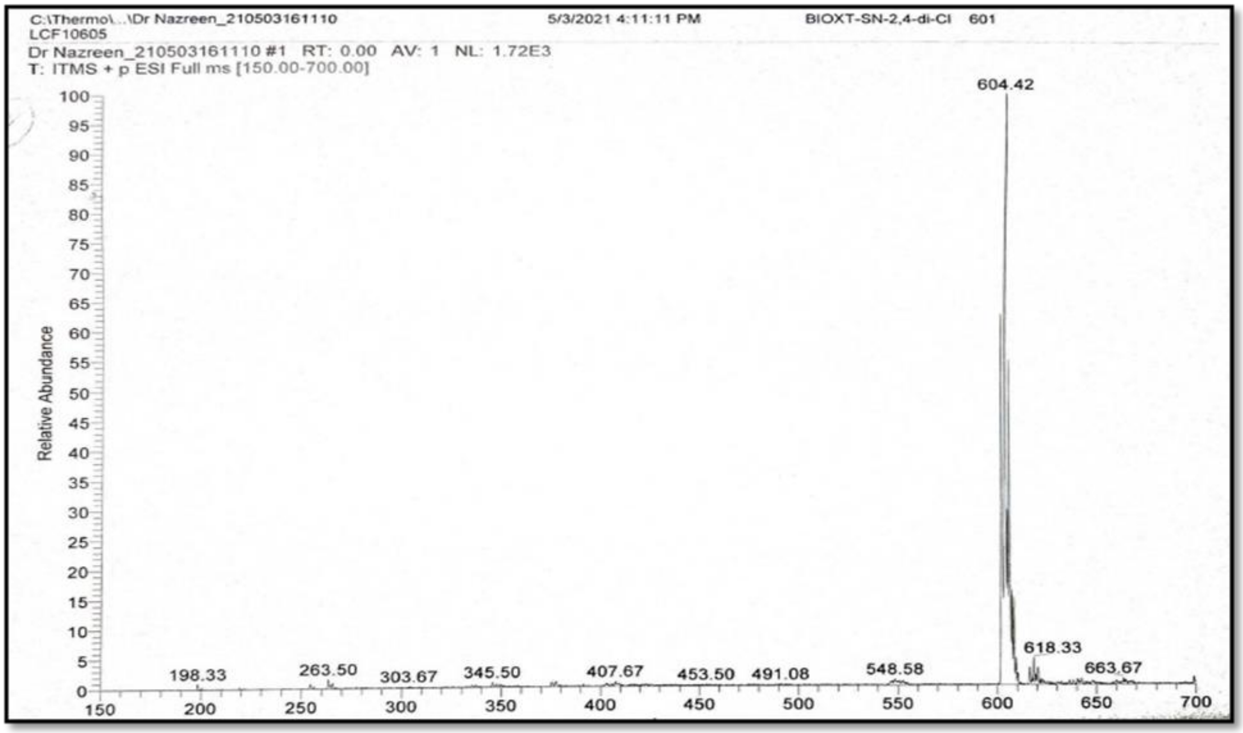

Figure S26: Mass of Compound 13

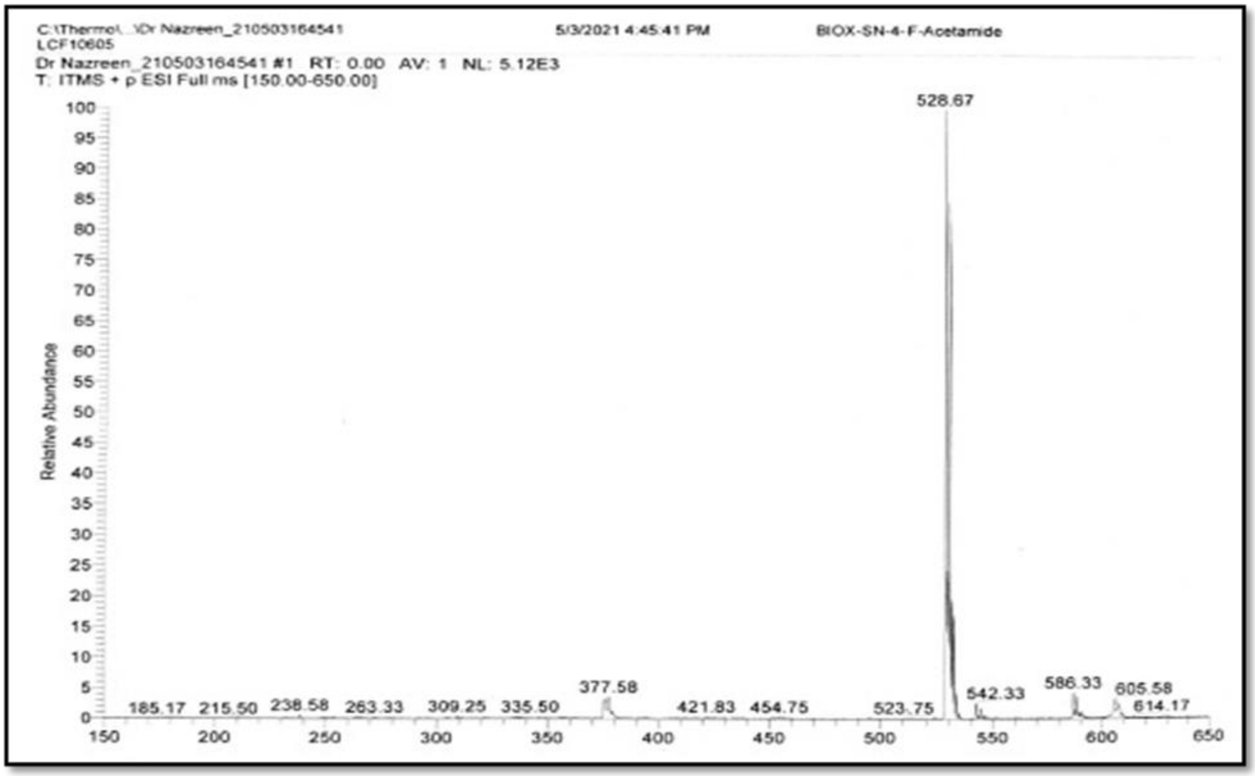

Figure S27: Mass of Compound 14

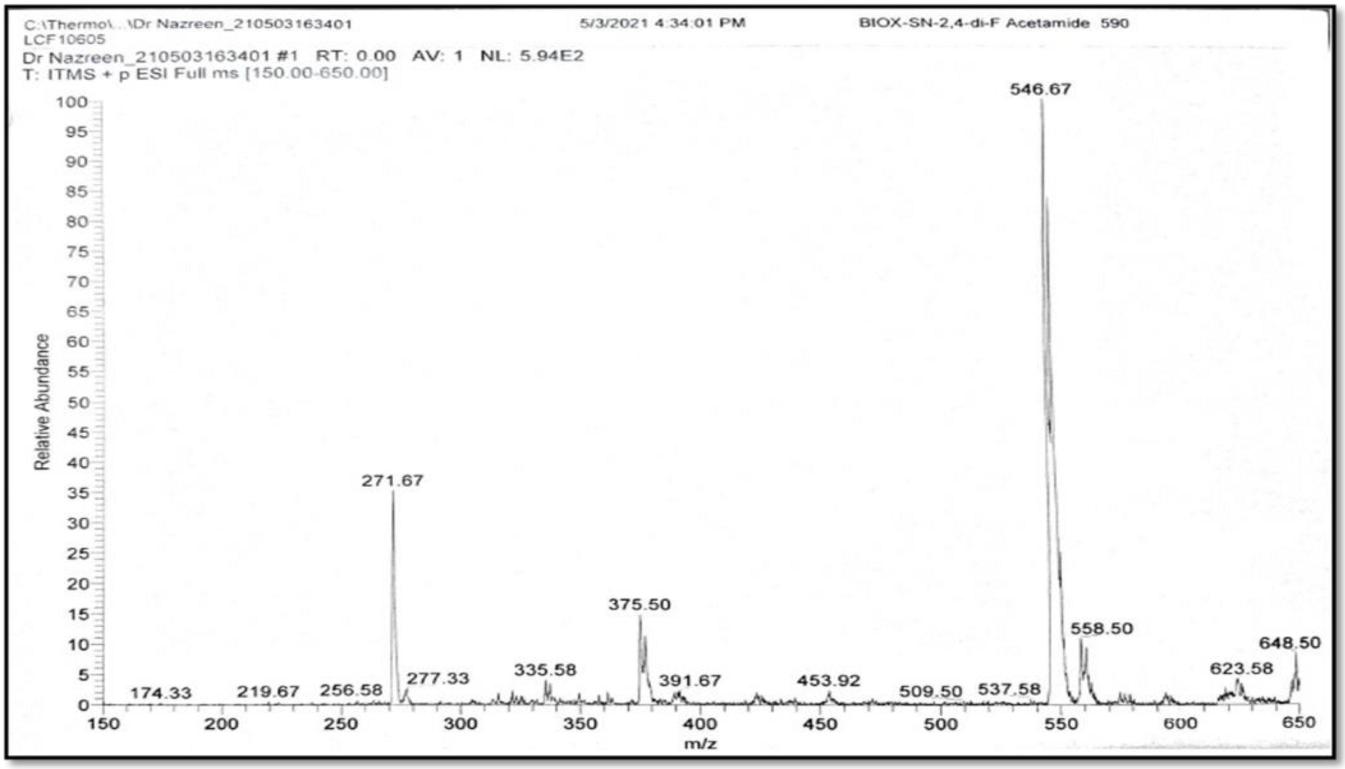

Figure S28: Mass of Compound 15

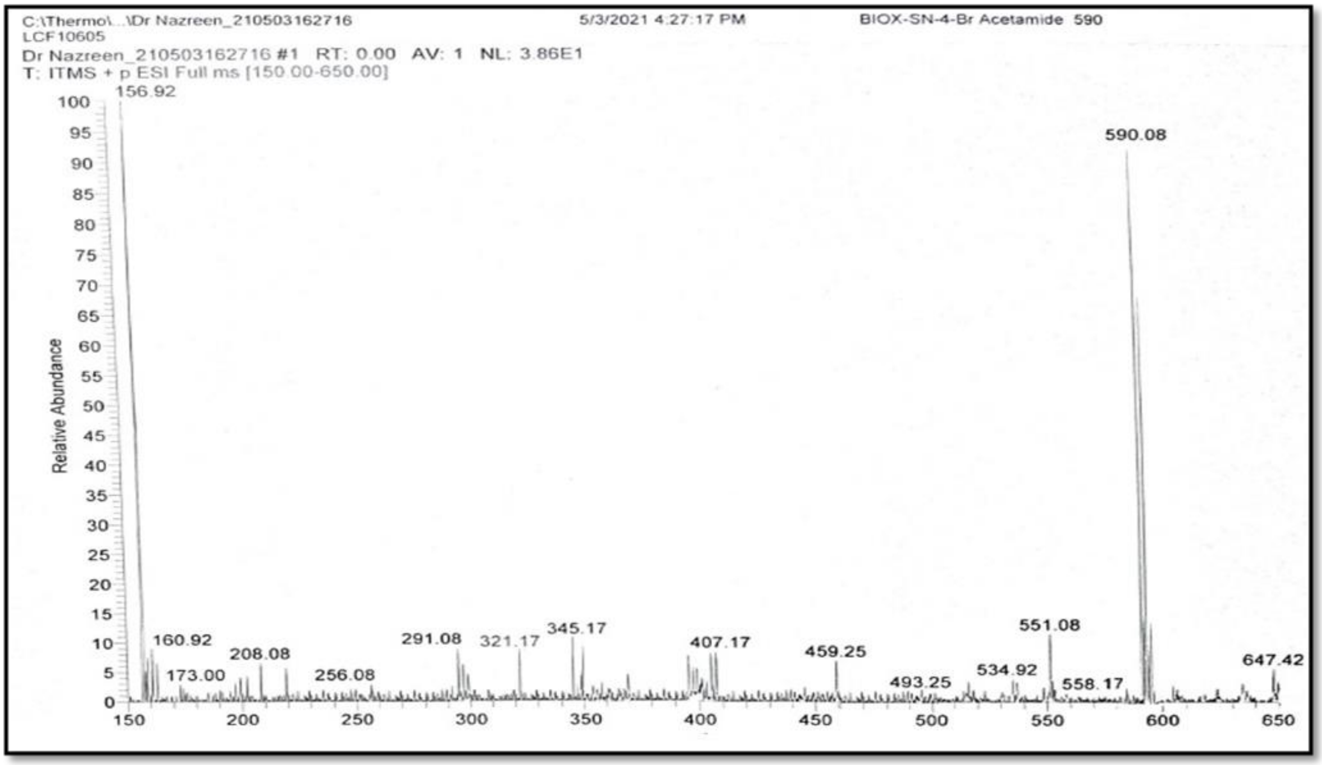

Figure S29: Mass of Compound 16

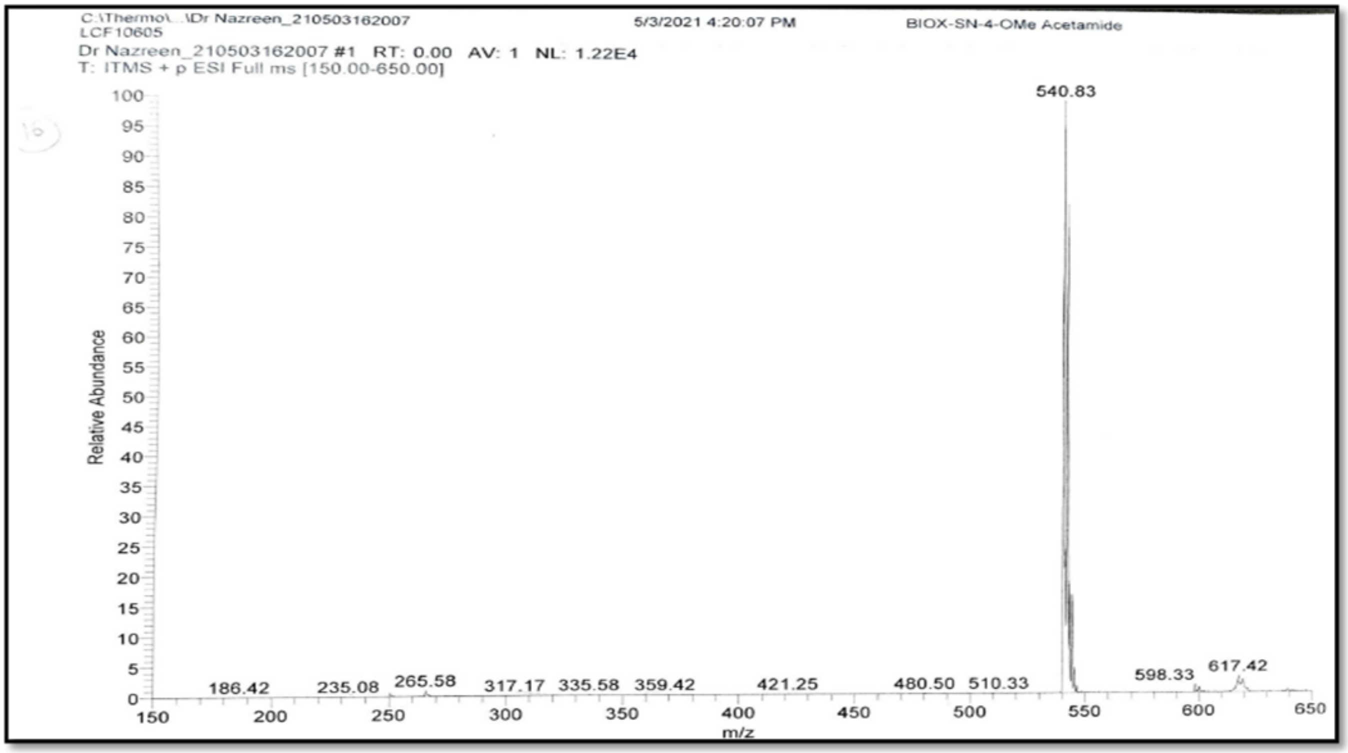

Figure S30: Mass of Compound 17

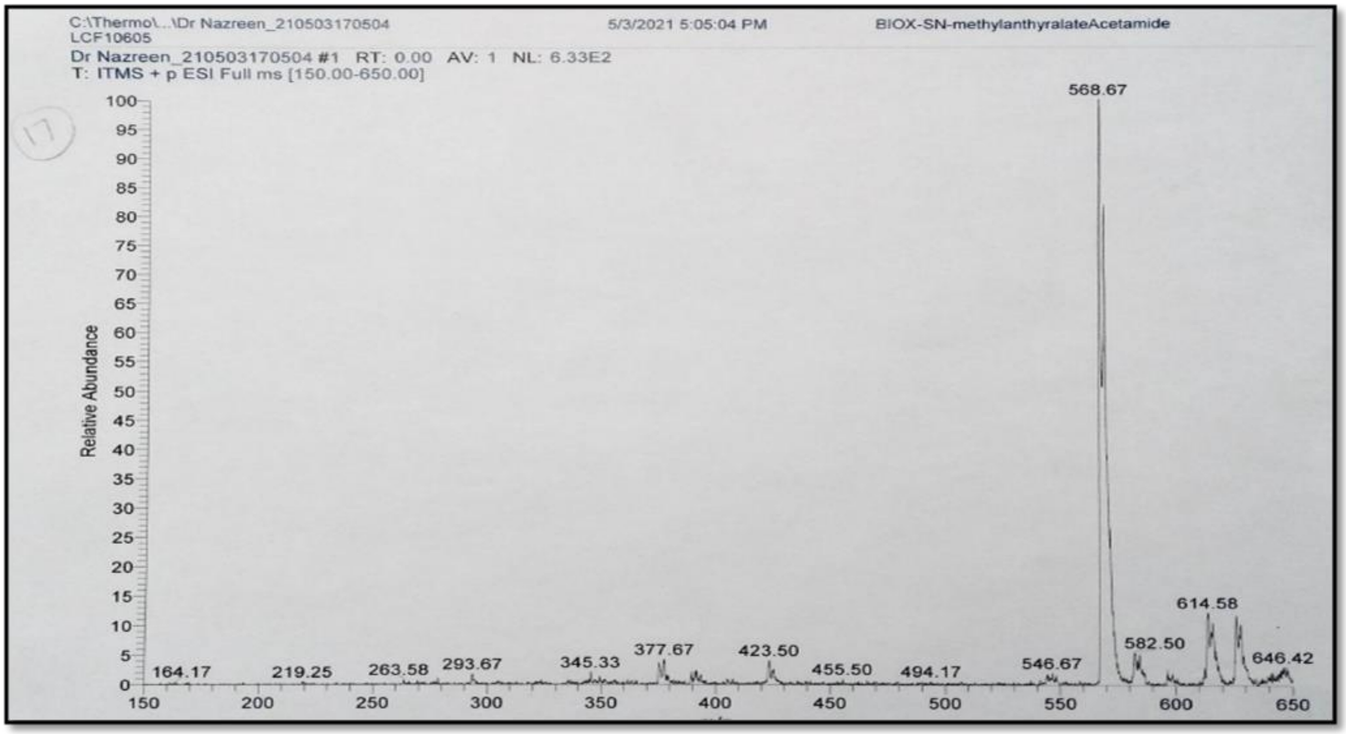

Supplement: Supplementary file 1 [file molecules-27-06899-s001.zip › molecules-1943750-supplementary.pdf]
